# Inter-Ethnic/Racial Facial Variations: A Systematic Review and Bayesian Meta-Analysis of Photogrammetric Studies

**DOI:** 10.1371/journal.pone.0134525

**Published:** 2015-08-06

**Authors:** Yi Feng Wen, Hai Ming Wong, Ruitao Lin, Guosheng Yin, Colman McGrath

**Affiliations:** 1 Paediatric Dentistry & Orthodontics, Faculty of Dentistry, The University of Hong Kong, Hong Kong SAR, China; 2 Department of Statistics & Actuarial Science, Faculty of Science, The University of Hong Kong, Hong Kong SAR, China; 3 Periodontology & Public Health, Faculty of Dentistry, The University of Hong Kong, Hong Kong SAR, China; Bournemouth University, UNITED KINGDOM

## Abstract

**Background:**

Numerous facial photogrammetric studies have been published around the world. We aimed to critically review these studies so as to establish population norms for various angular and linear facial measurements; and to determine inter-ethnic/racial facial variations.

**Methods and Findings:**

A comprehensive and systematic search of PubMed, ISI Web of Science, Embase, and Scopus was conducted to identify facial photogrammetric studies published before December, 2014. Subjects of eligible studies were either Africans, Asians or Caucasians. A Bayesian hierarchical random effects model was developed to estimate posterior means and 95% credible intervals (CrI) for each measurement by ethnicity/race. Linear contrasts were constructed to explore inter-ethnic/racial facial variations. We identified 38 eligible studies reporting 11 angular and 18 linear facial measurements. Risk of bias of the studies ranged from 0.06 to 0.66. At the significance level of 0.05, African males were found to have smaller nasofrontal angle (posterior mean difference: 8.1°, 95% CrI: 2.2°–13.5°) compared to Caucasian males and larger nasofacial angle (7.4°, 0.1°–13.2°) compared to Asian males. Nasolabial angle was more obtuse in Caucasian females than in African (17.4°, 0.2°–35.3°) and Asian (9.1°, 0.4°–17.3°) females. Additional inter-ethnic/racial variations were revealed when the level of statistical significance was set at 0.10.

**Conclusions:**

A comprehensive database for angular and linear facial measurements was established from existing studies using the statistical model and inter-ethnic/racial variations of facial features were observed. The results have implications for clinical practice and highlight the need and value for high quality photogrammetric studies.

## Introduction

International migration is occurring at an unprecedented pace in the contemporary world [1]. The past 50 years has witnessed a dynamic increase in the number of international migrants from 92 million in 1960 to 165 million in 2000 [1] and to 214 million in 2010 [2]; The number is estimated to reach 405 million in 2050 [2]. Therefore, it is increasingly important for professionals from various medical and dental specialties whose work involves correction of facial anomalies and achieving aesthetics to be aware of the differences in facial characteristics among ethnic/racial groups.

While inter-ethnic/racial facial variations have long been of interest to the general public, anthropologists, and medical and dental practitioners, studies providing solid evidence on this issue are surprisingly sparse. One of the most comprehensive studies by Farkas and colleagues [3] compared normative facial measurements of a North American white population with data from other regions in the world; however, the generalizability of this study is limited by its small sample size (only 30 males and 30 females) in each participating country. Moreover, the facial features investigated were limited to linear measurements/parameters, and all comparisons were made against the North American white population.

Apart from the direct anthropometric method used by Farkas and colleagues [3], several indirect anthropometric methods exist, e.g. cephalometry, photogrammetry, three-dimensional stereophotogrammetry and surface laser scanning [4,5]. Of these methods, photogrammetry provides unique advantages over other methods from several perspectives [4,5]. First, the measurements are not affected by tissue sensitivity and compressibility, which is ideal for soft tissue analysis. Second, the examination procedure is less uncomfortable from both the subjects’ and examiners’ side and subjects are examined free from radiation exposure. Third, permanent photographic archives allowed flexibility in selection of and objectivity in assessment of facial measurements. Furthermore, equipment for photogrammetry is portable, the examination procedure is time saving and the cost is relatively low [6]. In addition, reliability of photogrammetry proved to be excellent [6]. Therefore, despite the advanced anthropometric methods such as three-dimensional stereophotogrammetry, photogrammetry remains the optimal choice for large epidemiological studies aiming at establishing population norms [6], especially in developing countries where sophisticated equipment is not available.

Results from different anthropometric methods are not directly comparable [7,8]. To date, no meta-analysis of photogrammetric studies has been performed. To fill in this gap, we aimed to conduct a systematic review and apply a statistical model to establish database for population norms of various angular and linear facial measurements for Africans, Asians and Caucasians; and to determine inter-ethnic/racial facial variations.

## Methods

This review was conducted according to a predetermined protocol (S1 Text) and was reported in line with recommendations from the MOOSE (Meta-analysis Of Observational Studies in Epidemiology) guidelines (S2 Text) [9].

### Data sources and search strategies

We comprehensively searched the electronic databases of PubMed (1997 onward), ISI Web of Science (1956 onward), EMBASE (1947 onward) and Scopus (1995 onward) with no restrictions on language, dates or status of publication. The initial search was updated to 1^st^ December, 2014 using automatic e-mail alerts. One reviewer (YFW) developed the search strategy and conducted the initial search using controlled vocabularies and keywords. The search strategy for all four databases is available in S3 Text. Reference lists of articles that were identified in the screening process were also manually searched.

### Study selection

Two trained and calibrated reviewers (YFW and HMW) independently screened titles and abstracts of the identified records during the first round screening. In the second round screening, full texts of those records judged to be potentially eligible were retrieved and assessed for eligibility. Inter-reviewer agreement was assessed using Cohen’s *κ*. Discrepant opinions between the reviewers were resolved by discussion at the end of each round, and a senior author (CM) was consulted if consensus could not be reached.

This review sought to identify all facial photogrammetric studies regardless of the type of study design. We considered studies for inclusion if they recruited African, Asian or Caucasian subjects between 18 to 45 years old; adopted the well-established definitions of facial landmarks and measurements (S1 and S2 Tables) [10–12]; and if standard error (SE) could be extracted or estimated from the report. Studies were excluded if they recruited exclusively the following subjects: attractive/beautiful subjects; subjects with severe malocclusion, developmental craniofacial disfigurement, history of facial trauma/fracture or cosmetic surgery; or patients with systematic disorders known to affect craniofacial development. Furthermore, we required the reported measurements to be accurate to one decimal place for linear measurements in millimeters and angular measurements in degrees. We attempted to acquire missing information by E-mail enquiry of the studies’ correspondence author whenever needed.

### Data extraction

Study characteristics and demographics such as name of the first author, year of publication, study location, origin of the subjects, sample source, sample size, age range, and gender were extracted. We also extracted details of the photographic process including the subjects’ body position, head posture, occlusal position, lip/chin posture and the camera-subject distance.

We intended to extract 11 angular and 18 linear facial measurements that have the greatest clinical implications (S3 and S4 Tables). Measurements were recorded by mean and standard deviation (SD); conversions were made if confidence interval or SE was reported. Articles reporting on more than one population group were regarded as many separate studies as the number of heterogeneous populations they contained. Different articles investigating the same group of subjects were considered as one study.

Data extraction was performed by one reviewer (YFW) using a predefined piloted spreadsheet in Microsoft Excel 2013 and the results of extraction were then verified by a second reviewer (HMW). Discrepancies were resolved by consensus or further consultation of a third investigator (CM).

### Assessment of risk of bias

To ascertain the validity of each eligible study, risk of bias was assessed based on an instrument [13] that has been used in systematic reviews on craniofacial anthropometrics [4,5]. Further modifications of the instrument were made in view of potential sources of bias unique to photogrammetric studies [14]. We included 17 items assessing four domains of the eligible studies: study design, photo taking process, facial measurements and the appropriateness of statistical analysis (S5 Table).

Our criteria for risk of bias assessment is detailed in S6 Table. A score of 0, 0.5 or 1 was assigned to each item indicating free of bias, partially free of bias and subject to bias, respectively. In cases of inapplicable items, no scores were given. A score was calculated for each study by dividing the sum of item scores by the total number of applicable items. Studies with scores below 0.40 were considered as with low risk of bias. Two trained and calibrated reviewers (YFW and HMW) assessed the studies and a third reviewer (CM) resolved discrepancies.

### Statistical analysis

Despite our extensive literature search, data for several facial measurements were still sparse, especially when analyses were stratified by gender. In addition, while we rigorously followed the predefined inclusion and exclusion criteria during article screening, there were still varying degrees of risk of bias among the eligible studies. To fully utilize our extracted data, a Bayesian hierarchical random effects model was constructed, with contrasts established for pairwise comparisons among the ethnic/racial groups.

The multilevel modelling approach naturally applies a hierarchical structure to the extracted data where individual studies were nested within ethnicities/races that in turn were nested within the total population. In addition, the Bayesian approach to multilevel modelling has additional advantages of allowing for greater flexibility in modelling variability at different levels and enabling us to make direct probability statements [15,16]. In the Bayesian hierarchical model, ethnicity/race-specific estimates of a facial measurement were more model-driven when there was substantial uncertainty on the basis of a small number of studies, whereas for ethnicities/races with less uncertainty, the estimates were more data-driven [17].

S4 Text and S1 and S2 Figs details statistical models for each level of the hierarchy. In a single level notation, the overall model to estimate facial measurements from the *i* ^*th*^ study of the *j* ^*th*^ ethnicity/race is:

$$y_{ij}=\mu_{00}+\eta_{0j}+\zeta_{ij}+\epsilon_{ij}$$

where *μ*_00_ is the grand mean of the facial measurement across ethnicities/races, *η*_0*j*_ and *ζ*_*ij*_ represent ethnicity/race-specific and study-specific random effects that are normally distributed with mean 0 and between-ethnicity/race variance *τ* ^2^ and between-study variance *σ* ^2^, respectively, and *ϵ*_*ij*_ denotes sampling error for each individual study.

Non-informative priors were specified for *τ* and *σ* using the half-Cauchy distribution with the scale set to be 25. The grand mean *μ*_00_ was assigned a non-informative normal prior, i.e. *μ*_00_ ∼ *N*(0, 10^4^). Linear contrasts were constructed to explore inter-ethnic/racial variations of the measurements [18].

We fitted the Bayesian hierarchical model using the Markov chain Monte Carlo (MCMC) algorithm to generate samples of posterior distributions of all model parameters, including ethnicity/race-specific estimates of facial measurements and the linear contrasts. The analyses were performed separately for males and females. A facial measurement was meta-analysed only if there were data from two or three ethnicities/races with at least one of the ethnicities/races included two or more eligible studies. Estimates of the facial measurements were informed by posterior means and 95% credible intervals (CrIs) of the posterior distributions. Inter-ethnic/racial variations were explored at significance levels of 0.05 and 0.10 by examining whether 0 was included in the 95% and 90% CrIs of the linear contrasts, respectively. The 95% (90%) CrI was obtained by taking the 2.5^th^ (5^th^) and 97.5^th^ (95^th^) percentiles of the posterior distributions. The MCMC sampling algorithm was performed using the JAGS software (version 3.4.0) [19] on R version 3.1.1 (R Development Core Team, 2014) [20].

## Results

### Literature search

**Fig 1:**
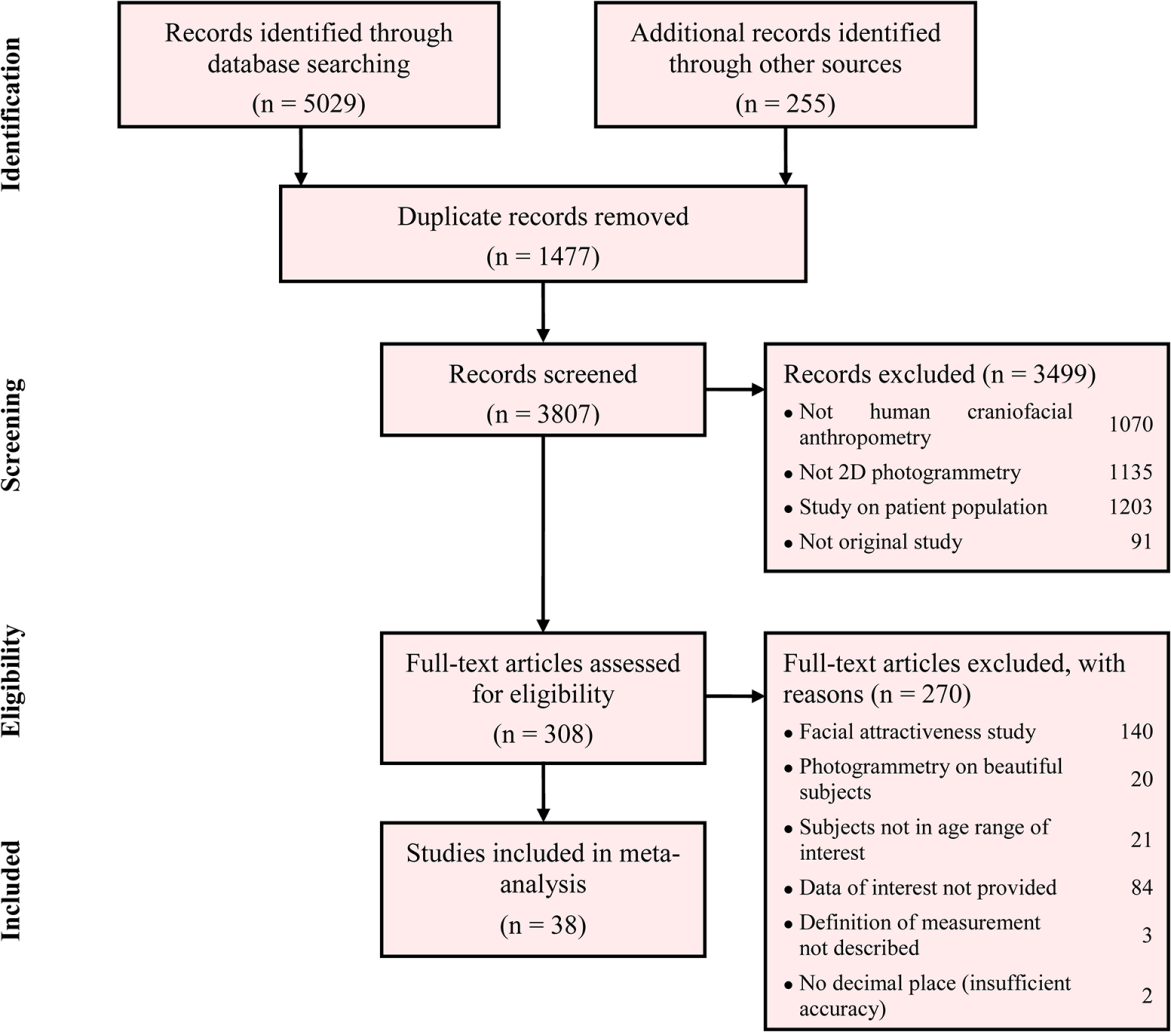
Flow diagram of study selection.

Fig 1 summarises the process of study identification and selection. We retrieved 3769 published original articles, abstracts, letters and reviews from the search of electronic databases and additional hand searching. After the first round study selection based on titles and abstracts (*κ* = 0.97), 308 potentially eligible articles were accessed for full-texts and underwent the second round study selection. Of these, 36 eligible articles [21–56] (*κ* = 0.95) that reported 38 studies were identified.

### Study characteristics

Characteristics of the eligible studies are detailed in Table 1. All studies had a cross-sectional design. The year of publication ranged from 1989 to 2014. One study was in Chinese, one in Korean, and the remaining 36 studies were in English. The studies involved 6686 subjects (male: 2944, female: 3742). Following Risch and colleagues’ ethnicity/race classification scheme on the basis of numerous population genetic surveys [57], subjects were considered as Africans if they were African Americans or Afro-Caribbeans originating from the sub-Saharan Africa; Asians if they were from China, Indochina (e.g. Cambodia, Malaysia, and Vietnam), Japan, Korea, the Philippines and Siberia in eastern Asia; and Caucasians if they were from Indian subcontinent, Middle East, North Africa with ancestry in Europe and West Asia. As a result, 1856 (27.8%) of the subjects were Africans (male: 1043; female: 813), 2720 (40.7%) were Asians (male: 1259; female: 1461), 2110 (31.5%) were Caucasians (male: 642; female: 1468).

**Table 1:** Characteristics of included studies.

Author, year	Study location	Origin of subjects	Sample size	Source of sample	Age (years)	Occlusal trait	Body position	Head posture	Occlusal position	Lip/chin posture	Camera-subject distance (m)
Akhter et al., 2013^21^	Bangladesh	Bangladeshi Christian Garo	100 (females)	..	25–45	..	Seated	..	..	..	1.2
Anibor et al., 2010a^22^	Nigeria	Igbo Nigerian	100 (50 males, 50 females)	..	18–25	Class Ⅰ occlusion	..	Natural head position	..	..	..
Anibor et al., 2010b^23^	Nigeria	Urhobo Nigerian	100 (50 males, 50 females)	..	18–25	Class Ⅰ occlusion	Sitting	Natural head position	..	..	..
Anibor et al., 2011^24^	Nigeria	Itsekiri Nigerian	100 (50 males, 50 females)	..	18–25	Class Ⅰ occlusion	..	..	..	..	..
Anic´-Miloševic et al., 2008 a^25^, b^26^	Croatia	Croatian	110 (52 males, 58 females)	A convenient sample of college students	23–28	Class Ⅰ occlusion	Standing	Natural head position	..	Repose	1.7
Bao et al., 1997^27^	China	Han Chinese	150 (75 males, 75 females)	College students, method of sampling not described	18–22	Normal occlusion	Standing	Frankfort plane parallel to the ground	Interocclusal rest space assumed	Relaxed and gently closed	Around 1.5
Chiu et al., 1992^28^	Hong Kong, China	Southern Chinese	59 (28 males, 31 females)	A convenient sample of volunteers from Guangdong province in southern China	19–30	Class Ⅰ occlusion	Standing	Natural head position	Maximum intercuspation	Relaxed	1.5
Choe et al., 2004^29^	US	Korean	72 (females)	A convenient sample of female Korean American volunteers	18–35	..	..	..	..	..	..
Eliakim-Ikechukwu et al., 2013 (Ibo Nigerian)^30^	Nigeria	Ibo Nigerian	276 (184 males, 92 females)	..	18–35	..	Seated	Anatomical position	..	..	1
Eliakim-Ikechukwu et al., 2013 (Yoruba Nigerian)^30^	Nigeria	Yoruba Nigerian	201 (106 males, 95 females)	..	18–35	..	Seated	Anatomical position	..	..	1
Etöz et al., 2008^31^	Turkey	White	173 in total (Sample size varies by measurement)	A convenient sample of white young adult volunteers	18–39	..	..	..	..	..	..
Ferdousi et al., 2013^32^	Bangladesh	Bangladeshi Christian Garo	100 (50 males, 50 females)	Bangladeshi Christian Garo in Dhaka city, method of sampling not described	25–45	..	Seated	Natural head position	..	..	1.0–1.5
Fernandez-Riveiro et al., 2002^33^	Spain	Galician Spanish	212 (50 males, 162 females)	A random sample of college students	18–20	..	Standing	Natural head position	..	Relaxed	..
Fernández-Riveiro et al., 2003^34^	Spain	Galician Spanish	275 (67 males, 208 females)	A random sample of college students	18–20	..	Standing	Natural head position	..	Relaxed	..
Gode et al., 2011^35^	Turkey	Turkish	Only control group included. 40 (20 males, 20 females)	Individuals satisfied with their facial appearance and were not considering plastic surgery, method of sampling not described	19–35	..	Ambiguous reporting	..	..	Closed	2
He et al., 2009^36^	China	Han Chinese	119 (56 males, 63 females)	A random sample of Han Chinese adults	18–25	Class Ⅰ occlusion	..	Frankfort plane parallel to the ground	..	..	1.6
Husein et al., 2010^37^	US	Indian	102 (females)	..	18–30	..	..	..	..	..	..
Kale-Varlk, 2008^38^	Turkey	Anatolian Turkish	111 (47 males, 64 females)	..	21–40	Skeletal Class Ⅰ	Standing	Natural head position	Centric occlusion	Repose	1.5
Lee et al., 1989^39^	Korea	Korean	120 (female)	..	18–45	..	Seated	Frankfort plane parallel to the ground	..	..	2
Lin et al., 2013^40^	Malaysia	Malaysian	102 (50 males, 52 females)	A random sample of college students	19–30	Class Ⅰ skeletal relationship	Standing	..	..	..	1
Loveday et al., 2011^41^	Nigeria	Igbo Nigerian	200 (100 males, 100 females)	A convenient sample of volunteers	18–35	..	..	Natural head position	..	Relaxed	1
Malkoç et al., 2009^42^	Turkey	Turkish	100 (46 males, 54 females)	A random sample of college students	19–25	Class Ⅰ occlusion	Standing	Natural head posture	..	Relaxed	..
Mostafa, et al., 2013 (25–35 yea-old group)^43^	Bangladesh	Bangladeshi Buddhist Chakma	70 (females)	A convenient sample of Bangladeshi Buddhist Chakma females	25–35	Regular dentition	..	..	..	Mouth closed naturally	1.2
Mostafa, et al., 2013 (35–45 yea-old group)^43^	Bangladesh	Bangladeshi Buddhist Chakma	30 (females)	A convenient sample of Bangladeshi Buddhist Chakma females	35–45	Regular dentition	..	..	..	Mouth closed naturally	1.2
Oghenemavwe et al., 2010^44^	Nigeria	Urhobo Nigerian	120 (60 males, 60 females)	A random sample of Urhobos	18–35	..	Standing	Natural head position	..	Relaxed	..
Osunwoke et al., 2014^45^	Nigeria	Nigerian	245 (160 males, 85 females)	A convenient sample of Nigerians in Okrika city	18–45	..	..	Natural head position	..	Relaxed	1
Ozdemir et al., 2009^46^	Turkey	Turkish	430 (149 males, 281 females)	A convenient sample of Turkish young adults	18–24	..	Standing	Natural head posture	..	Gently closed	2
Porter et al., 2001^47^	US	African	108 (females)	A convenient sample of female African American volunteers	18–30	..	..	..	..	..	..
Porter, 2004^48^	US	African	109 (males)	A convenient sample of male African American volunteers	18–30	..	..	..	..	..	..
Reddy et al., 2011^49^	India	North Indian	150 (78 males, 72 females)	A random sample of college students	18–25	Class Ⅰ dental relationship with pleasing profile	Standing	Natural head position	..	Relaxed	..
Sepehr et al., 2012^50^	Iran	Persian	107 (females)	A convenient sample of female Persian volunteers	18–40	..	..	..	..	..	..
Sim et al., 2000^51^	Singapore	Singaporean and Malaysian Chinese with southern Chinese ancestry	100 (females)	..	18–40	..	..	..	..	..	1
Song et al., 2007^52^	Korea	Korean	1282 (761 males, 521 females)	A convenient sample of Korean young adult volunteers	18–29	..	Standing	Frankfort plane parallel to the ground	..	..	1.5
Ukoha et al., 2012^53^	Nigeria	Igbo Nigerian	120 (males)	A random sample of students	18–28	..	..	Natural head position	..	Relaxed	1–1.5
Wamalwa et al., 2011 (Chinese)^54^	China	Chinese	156 (60 males, 96 females)	College students, method of sampling not described	20–26	Class Ⅰ occlusion	Standing	Natural head position	..	Relaxed	2.7
Wamalwa et al., 2011 (Kenyan)^54^	Kenya	Black Kenyan	177 (54 males, 123 females)	College students, method of sampling not described	20–26	Class Ⅰ occlusion	Standing	Natural head position	..	Relaxed	2.7
Wang et al., 2009^55^	Korea	Korean	21 (11 males, 10 females)	..	25–31	..	..	..	..	..	..
Yoo et al., 2013^56^	Korea	Korean	539 (218 males; 321 females)	A convenient sample of Korean young adult volunteers	18–29	..	Seated	Frankfort plane parallel to the ground	..	..	1.6

..: not reported.

### Risk of bias

Detailed risk of bias ratings are available in S5 Table. Of the 38 studies included in analysis, 23 (60.5%) were deemed low risk of bias, with the rest (39.5%) classed as high risk of bias. Scores of these studies ranged from 0.06 to 0.66. Over 70% of studies on Asians and 66.7% studies on Caucasians were of low risk of bias, whereas 58.3% of the African studies were subject to high risk of bias.

**Fig 2:**
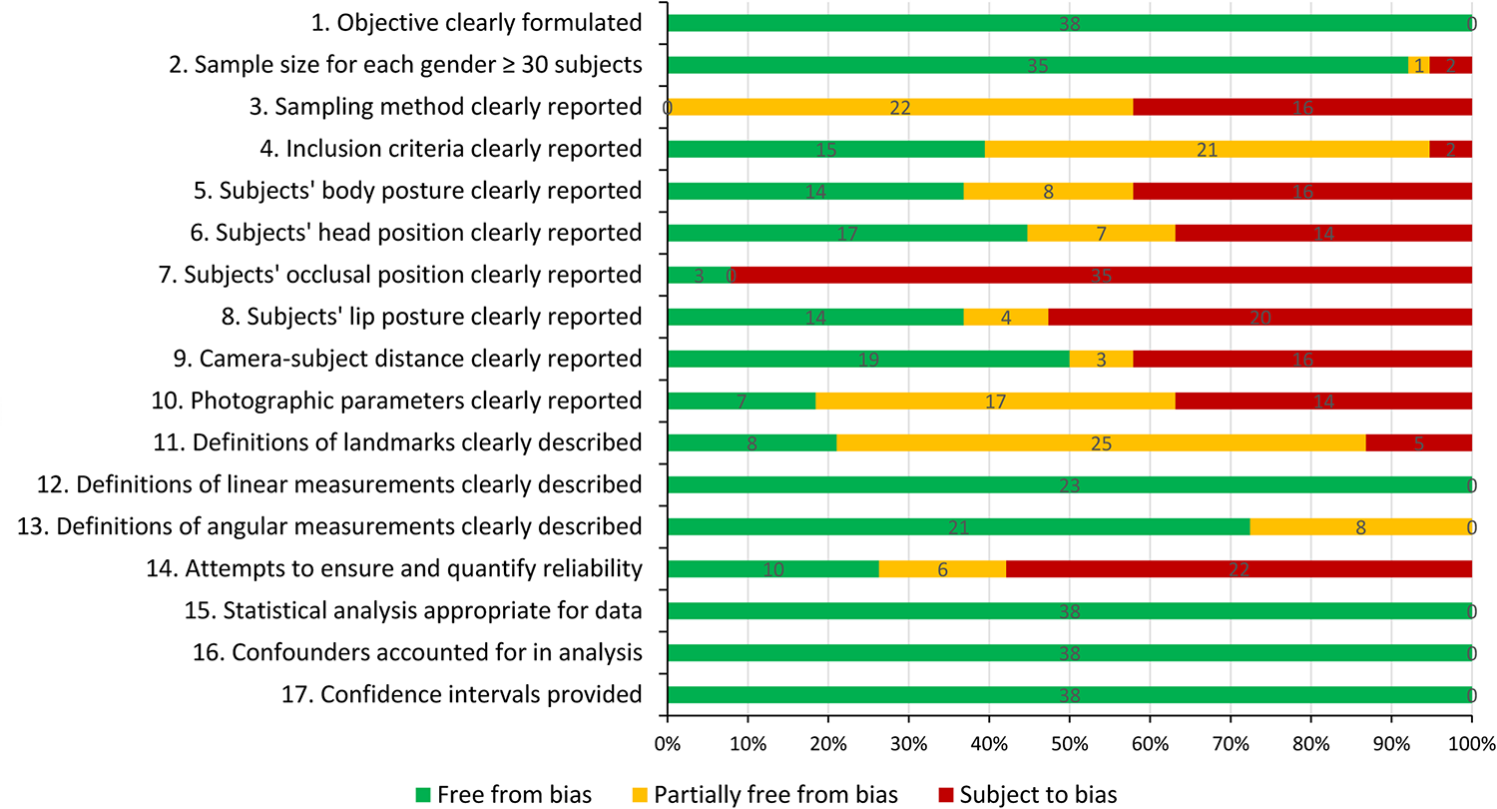
Risk of bias by item in the assessment instrument.

When each item in the instrument is assessed (Fig 2), sampling methods was found under-reported in most studies (57.9%). Regarding the photo taking process, most studies failed to adequately address the subjects’ body posture (63.2%), head position (55.3%) and lip posture (63.2%). Only three studies (8.9%) described the subjects’ occlusal position. Photographic parameters were reported in seven studies (18.4%). As to facial measurements, most studies defined facial landmarks by photo illustration (65.8%) and only eight studies provided written definitions. Measurement reliability was addressed in 16 studies (42.1%) and ten of them (26.3%) reported the reliability measure of method error.

### Ethnicity/race-specific population norm of facial measurements

Database for normative values of facial measurements was established at the ethnicity/race level by gender. Posterior means and corresponding 95% CrIs of the facial measurements were summarized in Tables 2 and 3. The number of studies and the number of subjects with which we obtained the posterior distributions were also recorded. Six measurements (angle of the medium facial third, angle of the inferior facial third, height of the upper face, height of the lower lip, vermilion height of the upper lip and vermilion height of the lower lip) were excluded from analysis due to small sample size (S4 Text).

**Table 2:** Ethnicity/race-specific population norm of facial measurements for males.

	African			Asian			Caucasian		
	No. of studies	No. of subjects	Mean (95% CrI)	No. of studies	No. of subjects	Mean (95% CrI)	No. of studies	No. of subjects	Mean (95% CrI)
**Nasofrontal angle**	11	1043	129.7 (126.5, 133.1)	4	202	133.7 (128.8, 138.8)	7	360	137.9 (133.6, 142.0)
**Nasal tip angle**	1	54	78.8 (70.6, 87.3)	2	116	79.7 (72.9, 86.6)	3	191	75.7 (70.1, 81.8)
**Nasolabial angle**	2	163	87.5 (76.6, 98.5)	6	280	94.7 (88.5, 100.6)	7	360	100.1 (94.0, 105.8)
**Nasofacial angle**	7	600	39.2 (36.8, 41.4)	1	75	31.9 (26.4, 38.3)	2	97	36.7 (33.0, 40.5)
**Nasomental angle**	6	430	124.1 (119.9, 128.6)	1	75	132.4 (123.7, 142.0)	5	247	129.4 (124.9, 133.9)
**Labiomental angle**	1	54	130.2 (122.0, 138.4)	3	185	134.8 (128.8, 140.4)	5	290	128.6 (124.3, 133.3)
**Angle of facial convexity**	1	54	168.5 (161.9, 175.3)	1	60	168.3 (161.5, 175.4)	6	340	167.8 (164.1, 171.4)
**Angle of total facial convexity**	1	54	145.4 (139.6, 150.3)	1	60	144.8 (139.3, 149.9)	3	191	141.5 (138.2, 145.4)
**Mentocervical angle**	9	774	89.2 (84.1, 94.6)	1	60	97.4 (85.7, 111.6)	3	191	94.3 (86.4, 102.8)
**Angle of the medium facial third**	..	..	..	..	..	..	..	..	..
**Angle of the inferior facial third**	..	..	..	..	..	..	..	..	..
**Width of the face**	1	109	138.1 (113.0, 162.6)	2	293	140.9 (118.7, 160.9)	1	149	130.7 (105.0, 157.1)
**Width of the mandible**	..	..	..	..	..	..	..	..	..
**Width of the nose**	2	269	37.9 (31.5, 44.2)	1	75	38.5 (31.2, 46.6)	2	169	37.9 (32.3, 43.5)
**Width of the mouth**	1	109	51.3 (37.8, 64.4)	2	836	48.4 (37.6, 59.3)	1	149	48.2 (35.0, 61.7)
**Height of forehead I**	1	109	58.0 (45.1, 71.8)	1	75	57.6 (45.1, 70.9)	4	271	55.1 (46.8, 63.6)
**Height of forehead II**	..	..	..	..	..	..	..	..	..
**Physiognomical height of the face**	..	..	..	2	293	189.2 (161.9, 215.9)	1	149	187.3 (154.8, 220.0)
**Height of the upper face**	..	..	..	..	..	..	..	..	..
**Height of the lower face**	1	109	72.2 (64.4, 80.0)	1	75	67.4 (59.7, 75.1)	4	271	69.4 (64.6, 73.9)
**Midface height**	1	109	62.9 (51.9, 73.7)	1	75	60.3 (48.6, 71.1)	4	271	65.5 (58.5, 72.1)
**Height of the nose**	2	269	47.9 (39.0, 57.1)	1	75	57.7 (45.4, 70.2)	4	271	51.1 (44.5, 57.6)
**Length of the nasal bridge**	..	..	..	..	..	..	..	..	..
**Nasal tip protrusion**	2	269	14.2 (7.7, 20.7)	..	..	..	2	102	12.6 (7.0, 18.1)
**Height of the upper lip**	..	..	..	1	75	21.9 (13.9, 29.8)	4	271	21.6 (16.9, 26.3)
**Height of the lower lip**	..	..	..	..	..	..	..	..	..
**Vermilion height of the upper lip**	..	..	..	..	..	..	..	..	..
**Vermilion height of the lower lip**	..	..	..	..	..	..	..	..	..
**Height of the mandible**	..	..	..	1	75	44.8 (33.2, 59.1)	2	201	47.4 (36.4, 57.6)

..: no data available for meta-analysis.

**Table 3:** Ethnicity/race-specific population norm of facial measurements for females.

	African			Asian			Caucasian		
	No. of studies	No. of subjects	Mean (95% CrI)	No. of studies	No. of subjects	Mean (95% CrI)	No. of studies	No. of subjects	Mean (95% CrI)
**Nasofrontal angle**	9	705	132.2 (126.2, 138.4)	6	436	139.3 (132.7, 145.9)	8	628	140.6 (134.6, 146.8)
**Nasal tip angle**	1	123	79.4 (70.7, 88.5)	2	159	81.4 (74.1, 88.7)	3	334	77.6 (71.3, 84.3)
**Nasolabial angle**	1	123	85.9 (69.5, 101.0)	9	619	94.2 (88.8, 99.8)	8	628	103.3 (96.9, 109.3)
**Nasofacial angle**	7	497	36.8 (34.5, 39.0)	1	75	31.5 (25.8, 37.2)	2	114	35.0 (31.2, 38.7)
**Nasomental angle**	5	310	126.2 (122.1, 130.4)	1	75	133.3 (125.6, 141.8)	5	264	129.5 (125.6, 133.4)
**Labiomental angle**	1	123	129.0 (120.1, 136.3)	3	223	133.4 (128.3, 138.5)	5	456	132.0 (127.9, 136.2)
**Angle of facial convexity**	1	123	170.8 (167.8, 173.6)	1	96	170.2 (167.5, 172.9)	6	506	168.2 (166.9, 169.6)
**Angle of total facial convexity**	1	123	147.4 (137.6, 155.9)	1	96	147.3 (137.2, 156.0)	3	334	141.0 (135.3, 148.1)
**Mentocervical angle**	8	620	88.9 (85.9, 92.0)	1	96	94.2 (87.3, 102.6)	3	334	91.1 (86.7, 95.6)
**Angle of the medium facial third**	..	..	..	..	..	..	..	..	..
**Angle of the inferior facial third**	..	..	..	..	..	..	..	..	..
**Width of the face**	1	108	134.2 (123.0, 145.1)	4	588	137.9 (130.3, 144.1)	2	383	123.3 (114.7, 134.6)
**Width of the mandible**	..	..	..	1	75	113.7 (77.7, 148.8)	2	383	105.5 (76.8, 136.1)
**Width of the nose**	2	193	34.6 (30.7, 38.1)	3	267	35.8 (32.8, 39.2)	4	510	35.1 (32.5, 37.6)
**Width of the mouth**	1	108	49.6 (43.5, 56.9)	4	788	46.9 (42.9, 50.7)	3	490	48.0 (43.8, 52.3)
**Height of forehead I**	1	108	55.0 (46.8, 63.9)	3	267	56.2 (50.4, 62.5)	6	730	52.8 (48.3, 57.5)
**Height of forehead II**	1	108	68.7 (62.7, 74.3)	1	72	72.8 (64.6, 77.8)	3	490	64.1 (60.7, 69.3)
**Physiognomical height of the face**	..	..	..	3	516	183.2 (173.8, 191.1)	3	483	171.7 (163.6, 181.2)
**Height of the upper face**	..	..	..	..	..	..	..	..	..
**Height of the lower face**	1	108	65.5 (59.9, 72.1)	2	147	64.6 (60.2, 69.4)	6	730	63.0 (60.0, 66.2)
**Midface height**	1	108	62.8 (54.8, 70.2)	3	267	63.1 (57.6, 68.4)	6	730	63.7 (59.5, 67.8)
**Height of the nose**	2	193	44.9 (38.4, 51.0)	3	267	50.7 (45.7, 55.9)	7	830	48.5 (45.1, 52.0)
**Length of the nasal bridge**	..	..	..	1	72	43.1 (32.4, 54.4)	2	209	42.2 (34.7, 49.7)
**Nasal tip protrusion**	1	85	13.1 (4.4, 20.2)	2	192	16.7 (10.9, 22.9)	4	429	15.3 (11.1, 19.5)
**Height of the upper lip**	..	..	..	2	195	20.9 (19.1, 22.7)	8	830	19.7 (18.7, 20.6)
**Height of the lower lip**	..	..	..	..	..	..	..	..	..
**Vermilion height of the upper lip**	..	..	..	..	..	..	..	..	..
**Vermilion height of the lower lip**	..	..	..	..	..	..	..	..	..
**Height of the mandible**	..	..	..	2	195	40.4 (33.0, 48.1)	2	339	42.2 (34.7, 49.1)

..: no data available for meta-analysis.

### Inter-ethnic/racial facial variations

**Fig 3:**
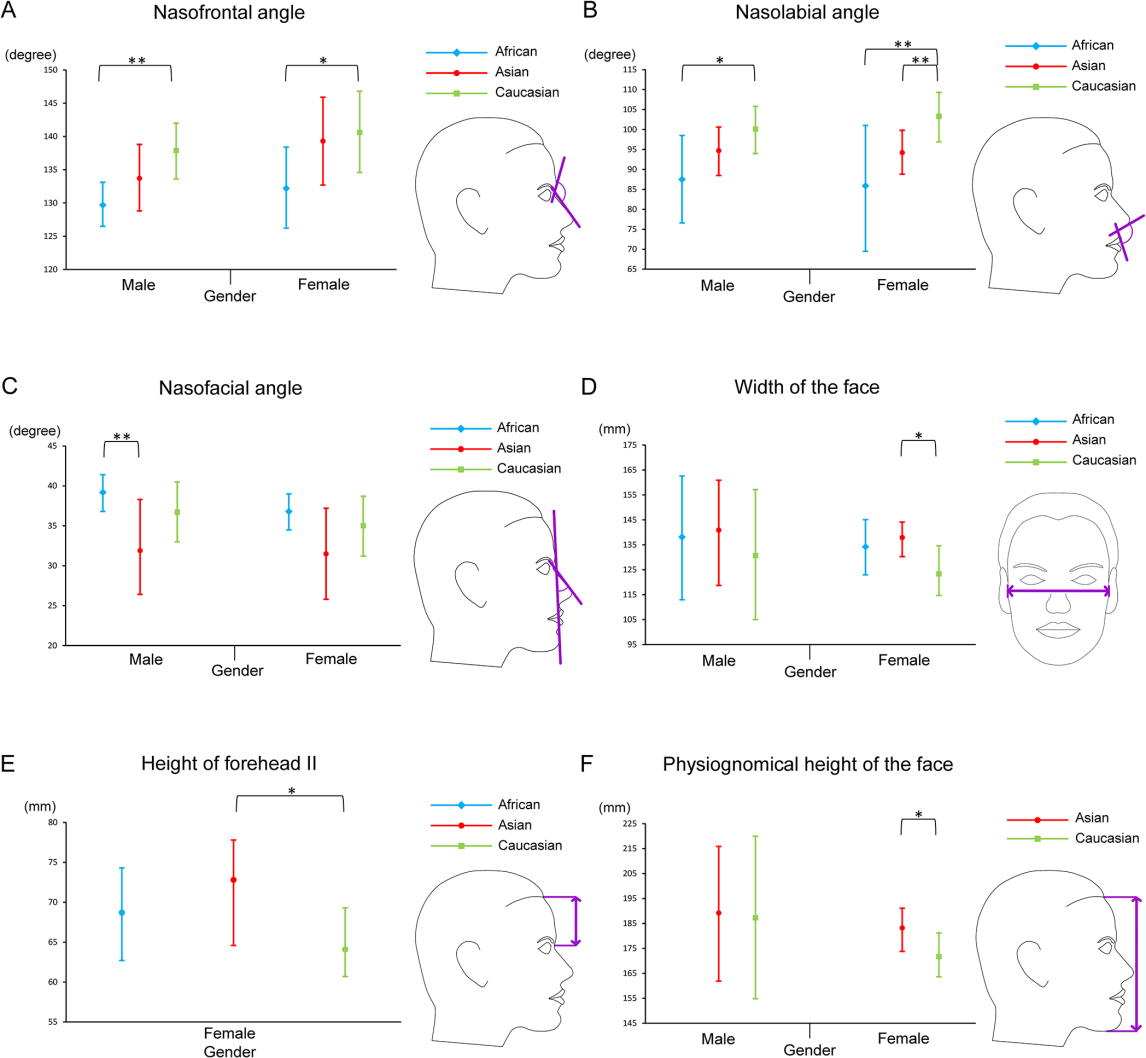
Facial measurements with significant inter-ethnic/racial variations by gender. (A) nasofrontal angle, (B) nasolabial angle, (C) nasofacial angle, (D) width of the face, (E) height of forehead II, (F) physiognomical height of the face. Error bars: 95% CrI. *: significantly different at 0.10 level of significance. **: significantly different at 0.05 level of significance.

Inter-ethnic/racial facial variations were summarized in Fig 3. Three measurements revealed inter-ethnic/racial variations at the significance level of 0.05. Nasofrontal angle was more obtuse in Caucasians than in Africans (posterior mean difference: 8.1°, 95% CrI: 2.2°–13.5°) among males. Nasolabial angle in Caucasian females was more obtuse than in African (17.4°, 0.2°–35.3°) and Asian (9.1°, 0.4°–17.3°) females. Asian males had on average more acute nasofacial angle compared to African males (7.4°, 0.1°–13.2°).

Additional inter-ethnic/racial facial variations were revealed when the statistical significance level was set at 0.10, which indicated a trend toward a significant difference. Caucasian females had larger nasofrontal angle than African females (8.5°, 90% CrI: 0.6°–15.9°). Nasolabial angle in males of Caucasians was on average 12.6° larger than in African males (0.8°–23.2°). As per linear facial measurements, Caucasian females had on average smaller width of the face (14.6°, 2.1°–23.2°), shorter height of forehead II (8.7°, 0.9°–12.8°) and shorter physiognomical height of the face (11.4°, 0.7°–20.2°) compared to Asian females.

## Discussion

This systematic review and meta-analysis is the first to collate all available photogrammetric studies to establish a comprehensive database for ethnicity/race-specific population norms of a variety of angular and linear facial measurements. Furthermore, this study for the first time comprehensively explored inter-ethnic/racial facial variations among the three major ethnic/racial groups. Our study provides strong evidence of inter-ethnic/racial variations as per nasofrontal angle, nasolabial angle and nasofacial angle. In addition, we observed substantial inter-ethnic/racial differences for linear measurements including width of the face, height of the forehead II and physiognomical height of the face.

Our meta-analysis updates results of an international anthropometric study [3] and a systematic review [58]. Compared with these studies, the meta-analysis adds to the literature by including both angular and linear facial measurements rather than being restricted to linear measurements related to the neoclassical canons [59]. Besides, our approach to investigating inter-ethnic/racial facial variations were more intuitive than relying on frequency distributions of arbitrarily defined categories [3] or focusing on the variance component of the measurements [58].

Ethnic/racial categorization in medical research is an issue of ongoing debate [57,60,61]. Despite the claim from some medical journals that ethnic/racial categorization is biologically meaningless [62,63], these discussions have been challenged due to a lack of solid scientific basis [57]. Before genetic and environmental determinants of facial characteristics are fully identified, ethnicity/race as a cruder surrogate factor to investigate facial variations remains a useful approach [57].

Our analysis of angular measurements revealed significant inter-ethnic/racial variations for nasofrontal angle, nasolabial angle and nasofacial angle. Nasofrontal and nasofacial angle are both affected by the position of nasion and nasal tip protrusion [12,64]. The smaller nasofrontal angle in African males compared to Caucasian males (posterior mean difference: 8.1°, 95% CrI: 2.2°–13.5°) and larger nasofacial angle in Africans compared to Asians (7.4°, 0.1°–13.2°) may be a reflection of the more inclined nasal bridge in Africans. Nasolabial angle is a critical determinant of nasal tip aesthetics [65]. The larger estimated nasolabial angle in Caucasian females indicates the prognathic feature of Africans and Asians [66]. As per linear facial measurements, our results suggest that width of the face and height of forehead II are significantly larger in Caucasian females than in Asian females, which are consistent with previous preliminary study [46] and systematic review [58]. While previous studies reported moderate inter-ethnic/racial variations regarding the nose [3,58], the present study failed to identify such differences.

The database established in this study provides normative range of facial measurements. Compared to the existing database [3], our database is more comprehensive in terms of the number of subjects used to derive the normative values and the more comprehensive coverage of facial features. Equipped with knowledge about this normal range, plastic and craniofacial surgeons are better informed in determining the amount of surgical corrections needed for a particular patient taking his/her ethnicity/race into consideration. This brings us closer to the ultimate goal of individualized treatment in plastic surgery. Besides, the database provides critical parameters for the manufacture of respirators and oxygen masks, whose design requires taking the consumers’ ethnicities/races into consideration. In addition, the results provide a platform for future genetic, nutritional and environmental studies to identify factors influencing facial morphology.

The strengths of this study rest on several aspects. First, the well-established definitions of landmarks and measurements [10,12] were complied, which ensured homogeneity of the measurements. Second, risk of bias was assessed following priori defined criteria (S6 Table) to enhance objectivity in assessment. Third, the Bayesian hierarchical model provides statistical advantages over traditional subgroup analysis in meta-analysis. The frequentist approach to meta-analysis yields 95% confidence intervals that are in fact narrower than the range of values they intended to cover [67]; besides, the no pooling nature of subgroup analysis tends to overestimate the variation among ethnicities/races [68]. Therefore, the frequentist approach to subgroup analysis tends to result in an inflated type I error rate compared to Bayesian hierarchical modelling. The type I error rate can be further increased when subgroups are pairwise compared post-hoc. In contrast, pairwise comparisons in Bayesian approach do not affect the rate of type I error since there is only one posterior distribution regardless of how comparisons are made [18].

There are several limitations in the current study. First, our meta-analysis inherits the limitations of original research. Since not all of the eligible studies were conducted as ethnicity/race-specific studies, subjects’ ethnicity/race had to be classified according to an external classification scheme. While the scheme proposed by Risch and colleagues [57] is well established, possibilities of ethnicity/race misclassification still could not be completely obviated. The issue could be further complicated by the increasing presence of mixed ethnicity/race. We recommend future photogrammetric studies defining subjects’ ethnicity/race in a more rigorous way by using methods such as ancestral mapping to facilitate inter-ethnic/racial comparisons. Second, we did not adjust our analyses for age or anthropometric indices such as body weight, height or body mass index since they were reported in none of the eligible studies. Possibilities for residual confounding cannot be excluded from our estimates. Third, there is heterogeneity among the eligible studies in terms of the subjects’ posturing, camera-object distance and photographic parameters. Risk of bias of the studies differed and a notably high percentage of African studies (58.3%) were with high risk of bias. We accounted for such heterogeneity by using random effects model in our analysis. However, it should be noted that the use of statistical model in our analyses should not overshadow the importance of a universally adopted photographic set-up. The most detailed descriptions of photogrammetric set-up come from Fernández-Riveiro and colleagues [33,34] and their method has been used by other studies [42]. We recommend its universal usage for future photogrammetric studies. Finally, despite the extensive literature search, there is still scarcity of data for several facial measurements. Estimates derived from a small amount of data may be subject to bias when applied to the population at large. Besides, scarcity of data results in substantial uncertainty in the ethnicity/race-specific estimates as revealed by the wide Bayesian credible intervals. In addition, six measurements were excluded from analysis due to insufficient data. To overcome the challenges of sparse data, we used the Bayesian approach to account for uncertainty in the hierarchical modelling, which proved to be more accurate than the frequentist approach, especially for small sample sizes [68,69]. Generalizability of our findings could be improved by inclusion of more high quality photogrammetric studies.

Our study provides a comprehensive database for various angular and linear facial measurements based on the best available photogrammetric studies. Significant inter-ethnic/racial variations were found for both angular and linear measurements. The results can provide a useful resource to guide research and clinical practice. This study also highlights the need for more high quality photogrammetric studies employing standardized photographic techniques; and preferably from a large randomized sample comprising different ethnic/racial groups.

## Supporting Information

S1 Fig Plot of the hierarchical structure. Purple, blue and green represent the first, second and third level of the hierarchy, respectively. “P” indicates the total number of ethnicities/races and “m” is the number of studies informing the first ethnicity/race.
(TIF)

S2 Fig Path diagram illustrating the Bayesian hierarchical model. The diagram is plotted borrowing Curran et al.’s path diagramming system [70]. The box represents the dependent variable. Triangles with number “1” inside is used to define the intercept term, and the subscript to “1” reflects specific levels of the hierarchical structure. Circles represent unobserved random coefficients. Solid arrows represent regression parameters. Purple, blue and green color represent the first, second and third level of the hierarchy, repsectively. We incorported distribution of random error terms for each level of the hierarchy using dash dot arrow. Unknown parameters and their prior distributions are illustrated in red with dot arrows.
(TIF)

S1 Table Definitions of anthropometric landmarks used in this study. (DOCX)

S2 Table Definitions of standard anthropometric measurements used in this study. (DOCX)

S3 Table Angular measurements extracted for meta-analysis. (DOCX)

S4 Table Linear measurements extracted for meta-analysis. (DOCX)

S5 Table Risk of bias of included studies. (DOCX)

S6 Table Criteria for risk of bias assessment. (DOCX)

S1 Text Protocol of the systematic review. (DOCX)

S2 Text Meta-analysis of Observational Studies in Epidemiology (MOOSE) Checklist. (DOCX)

S3 Text Detailed search strategy. (DOCX)

S4 Text Specification of the Bayesian hierarchical random effects model. (DOCX)
