# Supplementary figures and images for: Inter-Ethnic/Racial Facial Variations: A Systematic Review and Bayesian Meta-Analysis of Photogrammetric Studies
Source: PLoS One. 2015 Aug 6;10(8):e0134525. doi: 10.1371/journal.pone.0134525 (PMC4527668; doi:10.1371/journal.pone.0134525)

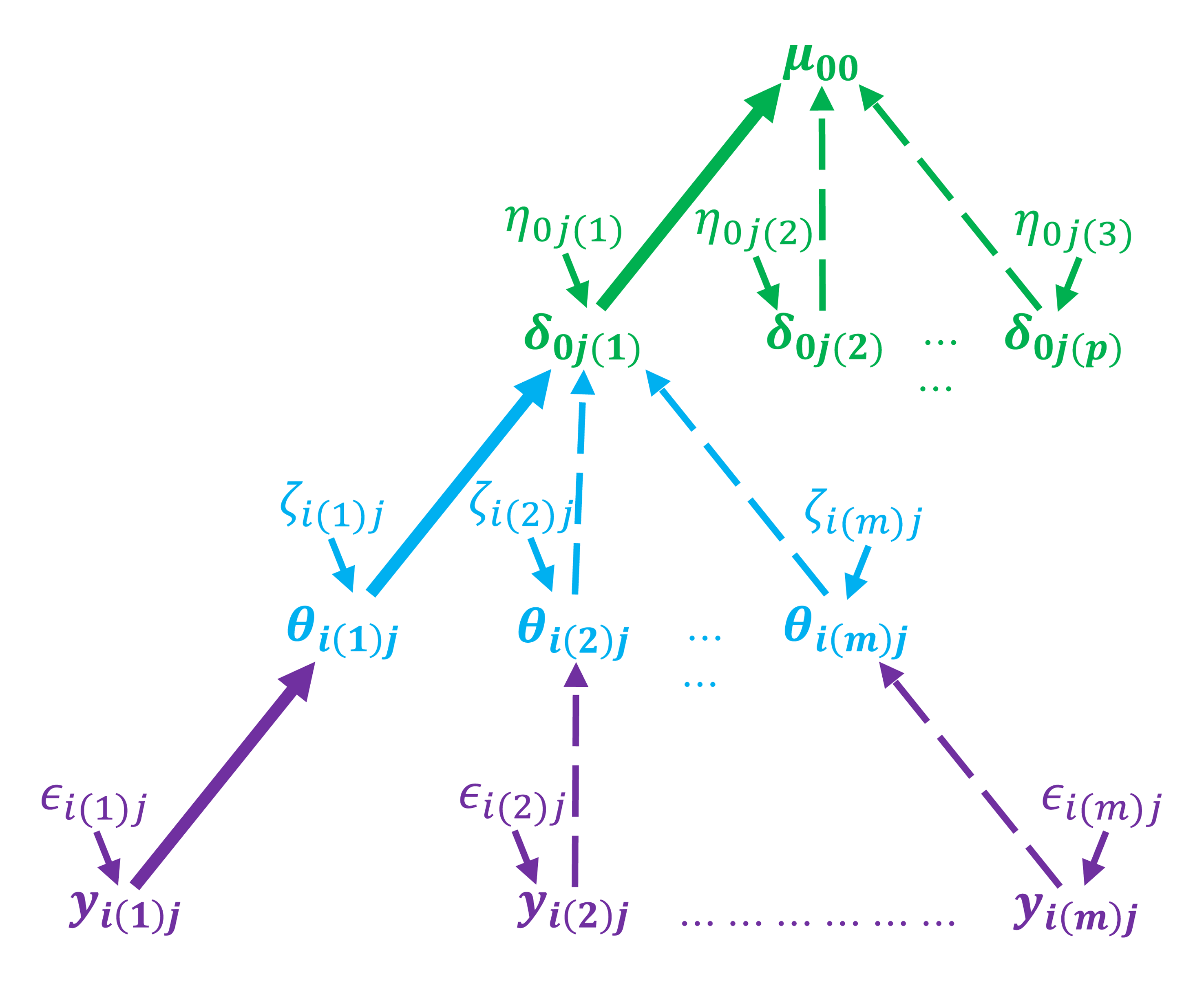

Supplement: S1 Fig — Purple, blue and green represent the first, second and third level of the hierarchy, respectively. “P” indicates the total number of ethnicities/races and “m” is the number of studies informing the first ethnicity/race. (TIF) [file pone.0134525.s001.tif]

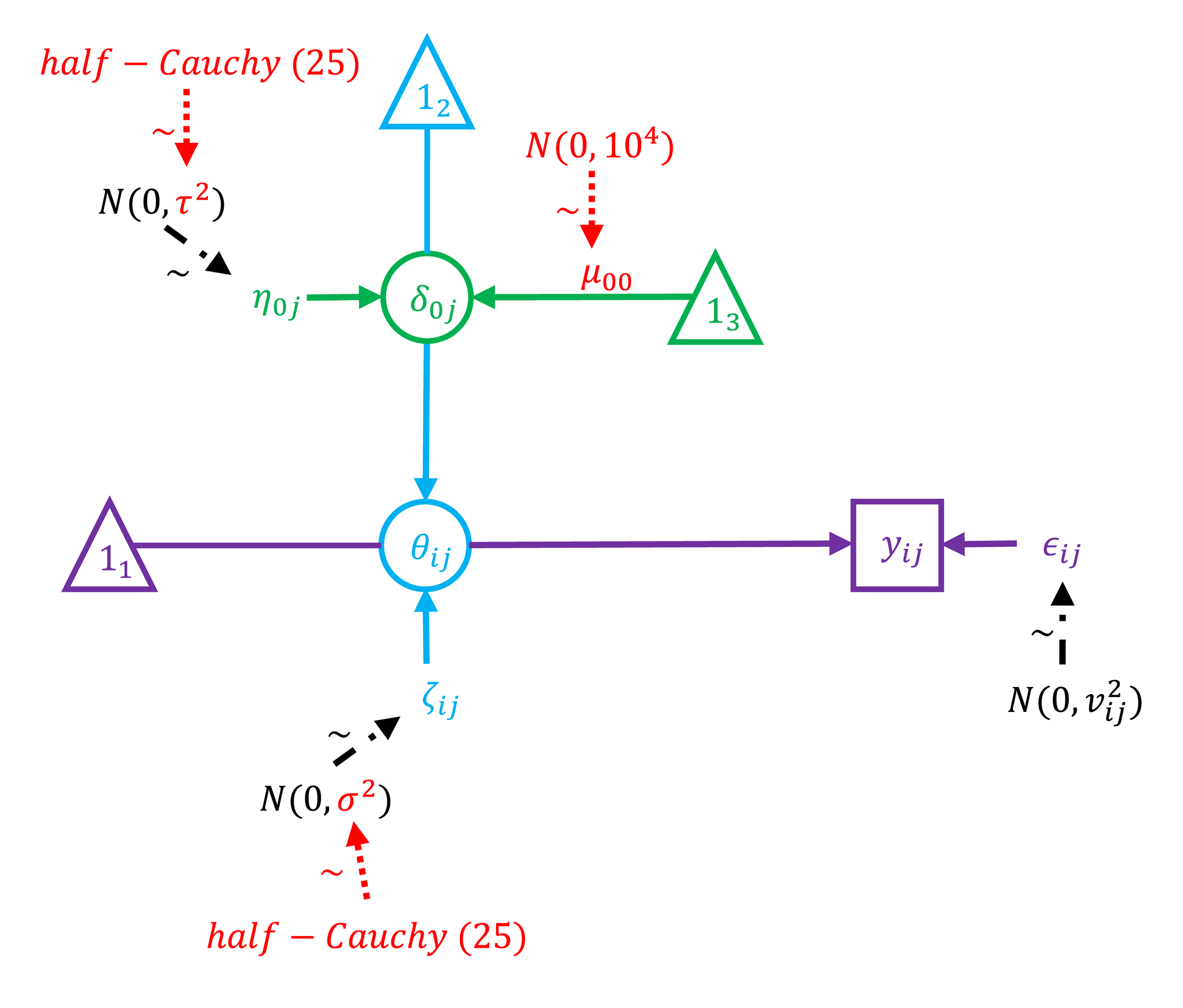

Supplement: S2 Fig — The diagram is plotted borrowing Curran et al.’s path diagramming system [70]. The box represents the dependent variable. Triangles with number “1” inside is used to define the intercept term, and the subscript to “1” reflects specific levels of the hierarchical structure. Circles represent unobserved random coefficients. Solid arrows represent regression parameters. Purple, blue and green color represent the first, second and third level of the hierarchy, repsectively. We incorported distribution of random error terms for each level of the hierarchy using dash dot arrow. Unknown parameters and their prior distributions are illustrated in red with dot arrows. (TIF) [file pone.0134525.s002.tif]
