# Supplementary material for: Inter-Ethnic/Racial Facial Variations: A Systematic Review and Bayesian Meta-Analysis of Photogrammetric Studies
Source: PLoS One. 2015 Aug 6;10(8):e0134525. doi: 10.1371/journal.pone.0134525 (PMC4527668; doi:10.1371/journal.pone.0134525)
Supplement: S1 Table — (DOCX) [file pone.0134525.s003.docx]

S1 Table. Definitions of anthropometric landmarks used in this study **(Naini, 2011 [1]).**

| **Landmark** | **Definition** |
| --- | --- |
| Trichion (tr) | The midline point at the junction of the hairline and forehead |
| Glabella (g) | The most prominent midline point of the forehead between the brow ridges |
| Nasion (n) | The point in the midline of the nasal radix and nasofrontal region |
| Zygion (zy) | The most lateral soft tissue point overlying each zygomatic arch |
| Tragion (t) | The notch in the superior margin of each tragus |
| Pronasale (prn) | The most prominent point on the nasal tip |
| Columella breakpoint (c') | The point at the mid-columella, where the columella takes a more horizontal course, extending posteriorly to subnasale |
| Alare (al) | The most lateral point on each alar contour |
| Subnasale (sn) | The deepest midline point where the base of the nasal columella meets the upper lip |
| Labrale superius (ls) | The midline point representing the mucocutaneous vermilion border of the upper lip |
| Stomion (sto) | The most anterior midline point of contact between the upper and lower lip |
| Cheilion (ch) | The point located at each lateral oral commissure |
| Labrale inferius (li) | The midline point representing the mucocutaneous vermilion border of the lower lip |
| Sublabiale (sl) | The midline point of greatest concavity on the facial contour of the lower lip between labrale inferius and soft tissue menton |
| Pogonion (pg) | The most prominent midline point of the soft tissue chin pad |
| Menton (me) | The most inferior midline point of the soft tissue chin |
| Gonion (go) | The most lateral point on the mandibular angle |
| Cervical point (c) | The innermost point between the submental region and the anterior surface of the neck, in the midsagittal plane |

**References**

1. Naini FB. Facial Aesthetics: Concepts and Clinical Diagnosis. West Sussex, UK: Wiley-Blackwell; 2011.
