# Supplementary material for: Inter-Ethnic/Racial Facial Variations: A Systematic Review and Bayesian Meta-Analysis of Photogrammetric Studies
Source: PLoS One. 2015 Aug 6;10(8):e0134525. doi: 10.1371/journal.pone.0134525 (PMC4527668; doi:10.1371/journal.pone.0134525)
Supplement: S2 Table — (DOCX) [file pone.0134525.s004.docx]

S2 Table. Definitions of standard anthropometric measurements used in this study **(Farkas, 1994 [1]; Powell, 1984 [2]).**

| **Measurement** | | **Definition** |
| --- | --- | --- |
| **Angular measurement** | |  |
|  | Nasofrontal angle | ∠g-n-prn |
|  | Nasal tip angle | ∠n-prn/c'-sn |
|  | Nasolabial angle | ∠c'-sn-ls |
|  | Nasofacial angle | ∠g-pg/n-prn |
|  | Nasomental angle | ∠n-prn-pg |
|  | Labiomental angle | ∠li-sl-pg |
|  | Angle of facial convexity | ∠g-sn-pg |
|  | Angle of total facial convexity | ∠g-prn-pg |
|  | Mentocervical angle | ∠c-me/g-pg |
|  | Angle of the medium facial third | ∠n-t-sn |
|  | Angle of the inferior facial third | ∠sn-t-me |
| **Linear measurement** | |  |
|  | Width of the face | zy-zy |
|  | Width of the mandible | go-go |
|  | Width of the nose | al-al |
|  | Width of the mouth | ch-ch |
|  | Height of foreheadⅠ | tr-g |
|  | Height of forehead Ⅱ | tr-n |
|  | Physiognomical height of the face | tr-me |
|  | Height of the upper face | n-sto |
|  | Height of the lower face | sn-me |
|  | Midface height | g-sn |
|  | Height of the nose | n-sn |
|  | Length of the nasal bridge | n-prn |
|  | Nasal tip protrusion | sn-prn |
|  | Height of the upper lip | sn-sto |
|  | Height of the lower lip | sto-sl |
|  | Vermilion height of the uppper lip | ls-sto |
|  | Vermilion height of the lower lip | sto-li |
|  | Height of the mandible | sto-me |

**References**

1. Farkas LG. Anthropometry of the Head and Face. 2nd ed. New York: Raven Press, Ltd.; 1994.

2. Powell N, Humphreus B. Proportions of the aesthetic face. New York: Thieme-Stratton Inc.; 1984.
