# Supplementary material for: Inter-Ethnic/Racial Facial Variations: A Systematic Review and Bayesian Meta-Analysis of Photogrammetric Studies
Source: PLoS One. 2015 Aug 6;10(8):e0134525. doi: 10.1371/journal.pone.0134525 (PMC4527668; doi:10.1371/journal.pone.0134525)
Supplement: S3 Table — (DOCX) [file pone.0134525.s005.docx]

# S3 Table. Angular measurements extracted for meta-analysis.

| **Author, year** | **Nasofrontal angle (∠g-n-prn)** | **Nasal tip angle (∠(n-prn/c'-sn))** | **Nasolabial angle (∠c'-sn-ls)** | **Nasofacial angle (∠(g-pg/n-prn))** | **Nasomental angle (∠n-prn-pg)** | **Labiomental angle (∠li-sl-pg)** | **Angle of facial convexity (∠g-sn-pg)** | **Angle of total facial convexity (∠g-prn-pg)** | **Mentocervical angle (∠(c-me/g-pg))** | **Angle of the medium facial third (∠n-t-sn)** | **Angle of the inferior facial third (∠sn-t-me)** |
| --- | --- | --- | --- | --- | --- | --- | --- | --- | --- | --- | --- |
| Anibor et al., 2010a [1] | Male: 128.0 (9.6); female: 140.2 (8.2) | .. | .. | Male: 40.1 (4.8); female: 37.8 (4.8) | Male: 124.4 (5.5); female: 126.9 (5.6) | .. | .. | .. | Male: 85.9 (12.7); female: 85.3 (8.8) | .. | .. |
| Anibor et al., 2010b [2] | Male: 132.0 (7.5); female: 100.56 (7.6) | .. | .. | Male: 39.6 (5.0); female: 37.4 (4.9) | Male: 127.0 (8.9); female: 127.4 (8.9) | .. | .. | .. | Male: 84.8 (6.5); female: 89.9 (8.2) | .. | .. |
| Anibor et al., 2011 [3] | Male:132.0 (11.4); female: 132.9 (9.8) | .. | .. | Male: 40.0 (4.3); female: 38.6 (5.2) | Male: 128.3 (4.7); female: 129.6 (4.98) | .. | .. | .. | Male: 82.6 (8.8); female: 86.94 (5.01) | .. | .. |
| Anic´-Miloševic et al., 2008 a [4], b [5] | Male: 136.38 (6.71); female: 139.11 (6.35) | .. | Male: 105.42 (9.52); female: 109.39 (7.84) | .. | Male: 130.47 (3.73); female: 130.19 (3.47) | Male: 129.26 (9.55); female: 134.50 (9.08) | Male: 168.78 (4.97); female: 169.05 (4.69) | .. | .. | .. | .. |
| Bao et al., 1997 [6] | Male: 131.69 (7.69); female: 138.90 (7.62) | .. | Male: 107.19 (8.27); female: 105.34 (8.95) | Male: 30.56 (3.27); female: 29.92 (2.56) | Male: 134.94 (4.43); female: 135.88 (3.48) | Male: 141.53 (12.11); female: 135.36 (7.48) | .. | .. | .. | .. | .. |
| Chiu et al., 1992 [7] | .. | .. | Male: 90.1 (14.6); female: 97.4 (10.5) | .. | .. | .. | .. | .. | .. | .. | .. |
| Choe et al., 2004 [8] | Female: 136.8 (6.4) | .. | Female: 92.1 (9.2) | Discarded | .. | .. | .. | .. | .. | .. | .. |
| Eliakim-Ikechukwu et al., 2013 (Population A) [9] | Male: 127.1 (7.46); female: 131.7 (5.08) | .. | .. | Male: 37.8 (6.10); female: 36.3 (3.55) | Discarded | .. | .. | .. | Male: 88.6 (4.48); female: 87.5 (6.23) | .. | .. |
| Eliakim-Ikechukwu et al., 2013 (Population B) [9] | Male: 127.9 (7.10); female: 134.3 (5.56) | .. | .. | Male: 37.3 (18.12); female: 35.5 (3.70) | Discarded | .. | .. | .. | Male: 85.9 (7.00); female: 85.6 (6.92) | .. | .. |
| Etöz et al., 2008 [10] | Discarded | .. | Discarded | .. | .. | .. | .. | .. | .. | .. | .. |
| Ferdousi et al., 2013 [11] | Male: 129.56 (7.96); female: 137.96 (4.79) | .. | Male: 91.28 (12.98); female: 91.92 (8.90) | Male: 40.27 (4.54); female: 38.67 (4.05) | Male: 129.75 (7.32); female: 132.79 (5.10) | .. | Male: 158.65 (12.17); female: 169.26 (4.43) | .. | .. | .. | .. |
| Fernández-Riveiro et al., 2003 [12] | Male: 138.57 (6.81); female: 141.98 (6.06) | Male: 72.6 (9.04); female: 76.28 (5.8) | Male: 105.2 (13.28); female: 107.57 (8.5) | .. | .. | Male: 130.75 (9.64); female: 131.45 (11.01) | Male: 168.2 (4.96); female: 167.0 (5.36) | Male: 139.9 (5.38); female: 139.2 (4.48) | Male: 79.85 (7.19); female: 84.18 (6.65) | Male: 28.9 (2.61); female: 28.2 (2.61) | Male: 36.8 (3.59); female: 36.2 (3.14) |
| Gode et al., 2011 [13] | Male: 143.3 (8.3); female: 140.9 (7.5) | .. | Male: 103.1 (5.3); female: 119.2 (9.7) | .. | Male: 132.2 (3.4); female: 128.7 (4.5) | .. | .. | .. | .. | .. | .. |
| He et al., 2009 [14] | Male: 138.15 (8.43); female: 147.71 (5.48) | Male: 77.11 (7.09); female: 78.36 (8.50) | Male: 98.50 (10.54); female: 100.05 (11.33) | .. | .. | .. | .. | .. | .. | .. | .. |
| Husein et al., 2010 [15] | Female: 138.2 (8.1) | .. | Female: 97.2 (10.6) | Discarded | .. | .. | .. | .. | .. | .. | .. |
| Kale-Varlk, 2008 [16] | Males: 139.5 (11.4); female: 142.2 (9.9) | .. | Male: 98.7 (13.7); female: 104.4 (12.7) | Male: 33.5 (2.5); female: 31.4 (3.4) | Male: 128.6 (3.4); female: 129.5 (3.2) | Male: 126.3 (4.3); female: 125.6 (4.0) | Male: 169.3 (5.3); female: 167.9 (4.7) | .. | .. | Male: 30.4 (3.9); female: 28.7 (3.6) | Male: 32.1 (4.0); female: 37.5 (4.7) |
| Lee et al., 1989 [17] | Female: 136.89 (6.49) | .. | Female: 85.36 (11.32) | .. | .. | .. | .. | .. | .. | .. | .. |
| Lin et al., 2013 [18] | .. | .. | Male: 92.99 (8.82); female: 95.04 (8.17) | .. | .. | Male: 130.44 (13.08); female: 130.73 (11.60) | .. | .. | .. | .. | .. |
| Loveday et al., 2011 [19] | Male: 130.18 (8.84); female: 134.29 (9.18) | .. | .. | Male: 39.92 (4.72); female: 38.06 (5.22) | Male: 112.92 (8.91); female: 117.25 (8.24) | .. | .. | .. | Male: 91.11 (2.25); female: 90.37 (1.28) | .. | .. |
| Malkoç et al., 2009 [20] | Male: 146.03 (8.19); female: 148.61 (6.66) | Male: 76.21 (6.72); female: 78.41 (9.17) | Male: 101.09 (10.19); female: 102.94 (10.43) | .. | .. | Male: 130.19 (8.50); female: 137.19 (10.93) | Male: 170.60 (6.15); female: 168.78 (5.44) | Male: 142.35 (5.36); female: 142.57 (5.29) | Male: 104.86 (9.86); female: 95.64 (7.74) | Male: 29.94 (2.39); female: 29.33 (2.58) | Male: 34.77 (2.61); female: 34.40 (2.80) |
| Oghenemavwe et al., 2010 [21] | Male: 117.75 (9.07); female: 127.85 (9.50) | .. | .. | Male: 40.77 (6.29); female: 35.60 (7.46) | Male: 121.95 (7.93); female: 126.55 (6.93) | .. | .. | .. | Male: 93.33 (3.27); female: 90.88 (3.58) | .. | .. |
| Osunwoke et al., 2014 [22] | Male: 134.13 (0.85); female: 137.68 (5.63) | .. | .. | .. | .. | .. | .. | .. | .. | .. | .. |
| Porter, 2004 [23] | Male: 126.9 (9.50) | .. | Male: 83.1 (13.78) | .. | .. | .. | .. | .. | .. | .. | .. |
| Reddy et al., 2011 [24] | Male: 136.71 (3.64); female: 144.33 (1.75) | Male: 75.09 (3.17); female: 75.35 (3.08) | Male: 102.32 (4.69); female: 101.50 (4.39) | .. | Male: 127.71 (1.97); female: 127.11 (1.81) | Male: 124.24 (4.56); female: 132.03 (4.75) | Male: 168.54 (3.23); female: 166.64 (4.09) | Male: 141.23 (2.76); female: 138.62 (2.82) | Male: 100.93 (3.66); female: 94.11 (5.86) | .. | .. |
| Sepehr et al., 2012 [25] | Discarded | .. | Discarded | Discarded | .. | .. | .. | .. | .. | .. | .. |
| Sim et al., 2000 [26] | Discarded | .. | Female: 87.8 (8.87) | Discarded | Discarded | .. | .. | .. | Discarded | .. | .. |
| Ukoha et al., 2012 [27] | Male: 134 (8.97) | .. | .. | Discarded | Male: 126 (6.11) | .. | .. | .. | Male: 92 (5.44) | .. | .. |
| Wamalwa et al., 2011 (Population A) [28] | Male: 132.44 (6.91); female: 137.97 (5.21) | Male: 79.63 (6.24); female: 79.37 (7.04) | Male: 85.59 (11.66); female: 80.77 (10.33) | .. | .. | Male: 129.52 (10.75); female: 126.04 (11.66) | Male: 169.12 (3.59); female: 171.26 (4.49) | Male: 146.12 (4.85); female: 148.70 (4.59) | Male: 91.59 (4.55); female: 91.02 (7.74) | .. | .. |
| Wamalwa et al., 2011 (Population B) [28] | Male: 138.32 (4.65); female: 145.76 (4.22) | Male: 84.51 (6.31); female: 87.10 (5.76) | Male: 97.24 (9.04); female: 98.13 (6.86) | .. | .. | Male: 135.84 (10.35); female: 136.39 (7.75) | Male: 168.57 (5.44); female: 170.50 (3.80) | Male: 145.35 (5.47); female: 148.60 (3.58) | Male: 102.17 (7.50); female: 96.81 (6.42) | .. | .. |
| Wang et al., 2009 [29] | Male: 126.0 (6.3); female: 133.6 (5.2) | .. | Male: 78.5 (11.1); female: 82.7 (12.7) | .. | .. | .. | .. | .. | .. | .. | .. |

Data extracted in Mean (SD).

..: not reported. Discarded: measurements were reported but excluded from meta-analysis due to unstandardized definitions.

**References**

1. Anibor E. Photometric facial analysis of the Ibo ethnic group in Nigeria. Arch Appl Sci Res. 2010a;2: 219-222.

2. Anibor E. Photometric facial analysis of the Urhobo ethnic group in Nigeria. Arch Appl Sci Res. 2010b;2: 28-32.

3. Anibor E. Photometric facial analysis of the Itsekiri ethnic group in Nigeria. Adv Appl Sci Res. 2011;2: 145-148.

4. Anic-Milosevic S, Lapter-Varga M, Slaj M. Analysis of the soft tissue facial profile by means of angular measurements. Eur J Orthod 2008a;30: 135-140.

5. Anic-Milosevic S, Lapter-Varga M, Slaj M. Analysis of the soft tissue facial profile of Croatians using of linear measurements. J Craniofac Surg. 2008b;19: 251-258.

6. Bao B, Yu S, Tan J, Cai Y, Tian W, Ye X, et al. The analysis of frontal facial soft tissue of normal native adult of han race of Guangdong province by using the computer assisted photogrammetric-system. Hua Xi Kou Qiang Yi Xue Za Zhi. 1997;15: 266-268.

7. Chiu C, Clark R. The facial soft tissue profile of the southern Chinese: prosthodontic considerations. J Prosthet Dent. 1992;68: 839-850.

8. Choe K, Sclafani A, Litner J, Yu G, Romo TI. The Korean American woman's face: anthropometric measurements and quantitative analysis of facial aesthetics. Arch Facial Plast Surg. 2004;6: 244-252.

9. Eliakim-Ikechukwu C, Ekpo A, Etika M, Ihentuge C, Mesembe O. Facial aesthetic angles of the Ibo and Yoruba ethnic groups of Nigeria. IOSR J Pharm Biol Sci. 2013;5: 14-17.

10. Etöz B, Etöz A, Ercan I. Nasal shapes and related differences in nostril forms: a morphometric analysis in young adults. J Craniofac Surg. 2008;19: 1402-1408.

11. Ferdousi M, Mamun A, Banu L, Paul S. Angular Photogrammetric Analysis of the Facial Profile of the Adult Bangladeshi Garo. Adv Anthropol. 2013;3: 188-192.

12. Fernández-Riveiro P, Smyth-Chamosa E, Suárez-Quintanilla D, Suárez-Cunqueiro M. Angular photogrammetric analysis of the soft tissue facial profile. Eur J Orthod. 2003;25: 393-399.

13. Gode S, Tiris F, Akyildiz S, Apaydin F. Photogrammetric analysis of soft tissue facial profile in Turkish rhinoplasty population. Aesthetic Plast Surg. 2011;35: 1016-1021.

14. He Z, Jian X, Wu X, Gao X, Zhou S, Zhong X. Anthropometric measurement and analysis of the external nasal soft tissue in 119 young Han Chinese adults. J Craniofac Surg. 2009;20: 1347-1351.

15. Husein OF, Sepehr A, Garg R, Sina-Khadiv M, Gattu S, Waltzman J,et al. Anthropometric and aesthetic analysis of the Indian American woman's face. J Plast Reconstr Aesthet Surg. 2010;63: 1825-1831.

16. Kale-Varlk S. Angular photogrammetric analysis of the soft tissue facial profile of Anatolian Turkish adults. J Craniofac Surg 2008;19: 1481-1486.

17. Lee D, Kim W, Chung C, Kim S, Baek S. Photogrammetric study on the face of adult Korean female. J Korean Soc Plast Reconstr Surg. 1989;16: 423-432.

18. Lin C, Shaari R, Alam M, Rahman S. Photogrammetric Analysis of Nasolabial Angle and Mentolabial Angle norm in Malaysian Adults. Bangladesh J Med Sci. 2013;12: 209-214.

19. Loveday O, Babatunde F, Isobo U, Sunday O, Ijeoma O. Photogrammetric analysis of soft tissue profile of the face of Igbos in Port Harcourt. Asian J Med Sci. 2011;3: 228-233.

20. Malkoc S, Demir A, Uysal T, Canbuldu N. Angular photogrammetric analysis of the soft tissue facial profile of Turkish adults. Eur J Orthod. 2009;31: 174-179.

21. Oghenemavwe E, Osunwoke A, Ordu S, Omovigho O. Photometric analysis of soft tissue facial profile of adult Urhobos. Asian J Med Sci. 2010;2: 248-252.

22. Osunwoke E, Omin E. Photometric facial analysis of soft tissue profile of Okrika adults. Annu Res Rev Biol. 2014;4: 1980-1987.

23. Porter J. The average African American male face: an anthropometric analysis. Arch Facial Plast Surg. 2004;6: 78-81.

24. Reddy M, Ahuja N, Raghav P, Kundu V, Mishra V. Computer-assisted angular photogrammetric analysis of the soft tissue facial profile of North Indian adults. J Indian Orthod Soc. 2011;45: 119-123.

25. Sepehr A, Mathew PJ, Pepper JP, Karimi K, Devcic Z, Karam AM. The Persian woman's face: a photogrammetric analysis. Aesthetic Plast Surg. 2012;36: 687-691.

26. Sim R, Smith J, Chan A. Comparison of the aesthetic facial proportions of southern Chinese and white women. Arch Facial Plast Surg. 2000;2: 113-120.

27. Ukoha U, Udemezue O, Oranusi C, Asomugha A, Dimkpa U, Nzeukwu L. Photometric facial analysis of the Igbo Nigerian adult male. Niger Med J. 2012;53: 240-244.

28. Wamalwa P, Amisi SK, Wang Y, Chen S. Angular photogrammetric comparison of the soft-tissue facial profile of Kenyans and Chinese. J Craniofac Surg. 2011;22: 1064-1072.

29. Wang J, Jang Y, Park S, Lee B. Measurement of aesthetic proportions in the profile view of Koreans. Ann Plast Surg. 2009;62: 109-113.
