# Supplementary material for: Inter-Ethnic/Racial Facial Variations: A Systematic Review and Bayesian Meta-Analysis of Photogrammetric Studies
Source: PLoS One. 2015 Aug 6;10(8):e0134525. doi: 10.1371/journal.pone.0134525 (PMC4527668; doi:10.1371/journal.pone.0134525)
Supplement: S4 Table — (DOCX) [file pone.0134525.s006.docx]

# S4 Table. Linear measurements extracted for meta-analysis.

| **Author, year** | **Width of the face (zy-zy)** | **Width of the mandible (go-go)** | **Width of the nose (al-al)** | **Width of the mouth (ch-ch)** | **Height of foreheadⅠ (tr-g)** | **Height of foreheadⅡ (tr-n)** | **Physiognomical height of the face (tr-me)** | **Height of the upper face (n-sto)** | **Height of the lower face (sn-me)** |
| --- | --- | --- | --- | --- | --- | --- | --- | --- | --- |
| Akhter et al., 2013 [1] | .. | .. | .. | .. | .. | .. | Female: 168.8 (11.1) | .. | .. |
| Anic´-Miloševic et al., 2008 a [2], b [3] | .. | .. | .. | .. | Male: 59.54 (4.60); female: 54.83 (4.28) | .. | .. | .. | Male: 71.16 (4.70); female: 63.47 (3.38) |
| Bao et al., 1997 [4] | Male: 137.97 (7.45); female: 136.82 (10.38) | Male: 117.92 (6.36); female: 119.18 (10.65) | Male: 39.37 (2.95); female: 37.66 (3.03) | Male: 45.48 (4.42); female: 44.52 (4.74) | Male: 59.13 (4.90); female: 61.26 (6.34) | .. | Male: 182.02 (9.81); female: 180.72 (13.03) | .. | Male: 65.76 (5.47); female: 63.24 (5.95) |
| Choe et al., 2004 [5] | Female: 139.0 (8.6) | .. | Female: 35.5 (3.4) | Female: 50.2 (4.0) | Female: 57.7 (6.4) | Female: 73.7 (7.0) | .. | .. | Female: 66.8 (5.6) |
| Etöz et al., 2008 [6] | .. | .. | Male (n=83): 35.7 (4.6); female (n=90): 31.8 (3.8) | .. | .. | .. | .. | .. | .. |
| Fernandez-Riveiro et al., 2002 [7] | .. | .. | .. | .. | Male: 45.33 (6.46); female: 45.24 (5.91) | .. | .. | .. | Male: 71.4 (5.69); female: 65.4 (4.33) |
| Gode et al., 2011 [8] | .. | .. | Male: 39.0 (4.0); female: 37.5 (3.1) | .. | Male: 59.2 (6.9); female: 59.1 (9.0) | .. | .. | .. | Male: 64.8 (6.5); female: 67.4 (7.3) |
| Husein et al., 2010 [9] | Female: 125.9 (10.1) | Female: 95.2 (11.0) | Female: 35.6 (3.3) | Female: 51.1 (5.2) | Female: 54.2 (6.3) | Female: 63.9 (7.4) | Female: 169.4 (13.3) | Female: 65.0 (4.2) | Female: 57.8 (7.5) |
| Lee et al., 1989 [10] | Female: 136.13 (4.45) | .. | Female: 36.18 (2.55) | Female: 43.99 (3.46) | Female: 53.26 (4.76) | .. | Female: 181.17 (6.02) | .. | .. |
| Mostafa, et al., 2013 (Population A) [11] | .. | .. | .. | .. | .. | .. | .. | .. | .. |
| Mostafa, et al., 2013 (Population B) [11] | .. | .. | .. | .. | .. | .. | .. | .. | .. |
| Osunwoke et al., 2014 [12] | .. | .. | Male: 33.3 (0.54); female: 29.9 (0.39) | .. | .. | .. | .. | .. | .. |
| Ozdemir et al., 2009 [13] | Male: 123.1 (13.0); female: 116.8 (11.4) | Male: 116.3 (12.6); female: 110.2 (16.5) | Male: 38.4 (4.4); female: 34.8 (2.9) | Male: 47.1 (5.4); female: 44.0 (4.1) | Male: 53.0 (8.6); female: 53.5 (6.7) | Male: 64.7 (9.7); female: 63.3 (6.9) | Male: 185.8 (18.4); female: 174.0 (18.3) | Male: 73.5 (8.2); female: 70.8 (6.5) | Male: 69.4 (8.1); female: 61.0 (6.0) |
| Porter et al., 2001 [14] | Female: 135.0 (5.51) | .. | Female: 38.0 (2.91) | Female: 51.6 (3.53) | Female: 55.7 (7.48) | Female: 68.9 (7.38) | .. | .. | Female: 67.0 (4.78) |
| Porter, 2004 [15] | Male: 139.5 (6.37) | .. | Male: 42.1 (2.92) | Male: 53.7 (3.76) | Male: 60.2 (7.93) | Male: 71.3 (8.14) | .. | .. | Male: 74.1 (5.32) |
| Sepehr et al., 2012 [16] | .. | .. | Female: 35.2 (3.0) | Female: 49.2 (4.3) | Female: 46.3 (14.7) | Female: 65.0 (19.8) | .. | Female: 70.1 (11.0) | Female: 61.5 (4.8) |
| Song et al., 2007 [17] | .. | .. | .. | Male: 50.0 (3.8); female: 46.7 (3.6) | .. | .. | .. | .. | .. |
| Yoo et al., 2013 [18] | Male: 150.6 (6.4); female: 142.5 (5.4) | .. | .. | .. | .. | .. | Male: 198.4 (9.1); female: 190.2 (7.9) | .. | .. |

(cont’d)

| **Author, year** | **Midface height (g-sn)** | **Height of the nose (n-sn)** | **Length of the nasal bridge (n-prn)** | **Nasal tip protrusion (sn-prn)** | **Height of the upper lip (sn-sto)** | **Height of the lower lip (sto-sl)** | **Vermilion height of the upper lip (ls-sto)** | **Vermilion height of the lower lip (sto-li)** | **Height of the mandible (sto-me)** |
| --- | --- | --- | --- | --- | --- | --- | --- | --- | --- |
| Akhter et al., 2013 [1] | .. | Female: 45.3 (3.6) | .. | .. | .. | .. | .. | .. | .. |
| Anic´-Miloševic et al., 2008 a [2], b [3] | Male: 67.94 (4.35); female: 64.69 (3.39) | Male: 53.80 (2.74); female: 50.27 (2.65) | .. | Male: 10.67 (1.72); female: 11.59 (1.91) | Male: 23.55 (2.64); female: 20.57 (2.01) | Male: 18.92 (2.29); female: 17.67 (1.73) | Male: 8.39 (1.29); female: 8.52 (1.35) | Male: 8.67 (1.62); female: 8.60 (1.35) | Male: 47.60 (3.00); female: 42.90 (2.48) |
| Bao et al., 1997 [4] | Male: 57.32 (4.41); female: 56.31 (5.34) | Male: 61.76 (4.52); female: 56.80 (3.81) | .. | .. | Male: 22.01 (2.32); female: 20.44 (1.83) | .. | .. | .. | Male: 44.07 (3.12); female: 38.22 (2.60) |
| Choe et al., 2004 [5] | Female: 67.9 (5.0) | Female: 51.8 (4.4) | Female: 43.5 (3.9) | Female: 19.6 (1.4) | .. | .. | .. | .. | .. |
| Etöz et al., 2008 [6] | .. | .. | Male (n=83): 45.4 (7.3); female (n=90): 42.7 (4.7) | Male (n=81): 14.8 (2.7); female (n=86): 15.4 (2.8) | .. | .. | .. | .. | .. |
| Fernandez-Riveiro et al., 2002 [7] | Male: 72.1 (4.88); female: 68.7 (4.66) | Male: 52.53 (4.12); female: 49.86 (3.7) | .. | Male: 11.6 (2.21); female: 11.1 (1.7) | Male: 23 (2.6); female: 21.43 (1.83) | Male: 19.01 (2.49); female: 17.48 (1.93) | Male: 7.27 (1.65); female: 7.43 (1.39) | Male: 8.36 (1.78); female: 8.59 (1.52) | .. |
| Gode et al., 2011 [8] | Male: 61.9 (3.1); female: 61.6 (5.2) | Male: 45.6 (4.1); female: 46.5 (3.0) | .. | .. | Male: 17.8 (3.2); female: 18.0 (2.9) | .. | .. | .. | .. |
| Husein et al., 2010 [9] | Female: 58.1 (5.5) | Female: 45.6 (3.5) | Female: 39.2 (3.9) | Female: 19.1 (2.0) | Female: 18.6 (3.2) | .. | Female: 8.3 (1.0) | Female: 10.1 (1.3) | .. |
| Lee et al., 1989 [10] | Female: 64.43 (3.64) | Female: 46.79 (3.55) | .. | Female: 16.03 (1.67) | Female: 21.70 (1.52) | .. | .. | .. | Female: 41.76 (2.43) |
| Mostafa, et al., 2013 (Population A) [11] | .. | .. | .. | .. | Female: 19.6 (1.9) | Female: 14.6 (1.9) | Female: 7.6 (1.2) | Female: 9.9 (1.6) | .. |
| Mostafa, et al., 2013 (Population B) [11] | .. | .. | .. | .. | Female: 20.2 (2.6) | Female: 15.0 (2.1) | Female: 7.3 (1.6) | Female: 9.6 (2.0) | .. |
| Osunwoke et al., 2014 [12] | .. | Male: 41.8 (0.74); female: 38.2 (0.49) | .. | Male: 12.2 (0.26); female: 10.7 (0.23) | .. | .. | .. | .. | .. |
| Ozdemir et al., 2009 [13] | Male: 63.7 (7.5); female: 61.4 (5.4) | Male: 51.9 (7.5); female: 51.7 (5.8) | Discarded | .. | Male: 21.6 (3.3); female: 19.3 (2.9) | Male: 19.5 (3.5); female: 17.5 (2.3) | Male: 5.3 (1.3); female: 5.1 (1.1) | Male: 9.1 (2.4); female: 9.0 (1.8) | Male: 47.8 (6.2); female: 42.1 (7.0) |
| Porter et al., 2001 [14] | Female: 62.0 (4.16) | Female: 48.0 (4.16) | .. | .. | .. | .. | .. | .. | .. |
| Porter, 2004 [15] | Male: 62.4 (4.38) | Male: 50.6 (3.97) | Male: 45.4 (4.38) | Male: 17.2 (1.77) | .. | .. | .. | .. | .. |
| Sepehr et al., 2012 [16] | Female: 69.1 (4.8) | Female: 50.8 (4.3) | Female: 44.1 (4.0) | Female: 19.7 (1.8) | Female: 19.0 (2.4) | .. | Female: 7.7 (1.4) | Female: 9.5 (1.7) | .. |
| Song et al., 2007 [17] | .. | .. | .. | .. | .. | .. | .. | .. | .. |
| Yoo et al., 2013 [18] | .. | .. | .. | .. | .. | .. | .. | .. | .. |

Data extracted in Mean (SD).

..: not reported. Discarded: measurements were reported but excluded from meta-analysis due to unstandardized definitions.

**References**

1. Akhter Z, Banu M, Alam M, Hossain S, Nazneen M. Photo-anthropometric study on face among Garo adult females of Bangladesh. Bangladesh Med Res Counc Bull. 2013;39: 61-64.

2. Anic-Milosevic S, Lapter-Varga M, Slaj M. Analysis of the soft tissue facial profile by means of angular measurements. Eur J Orthod 2008a;30: 135-140.

3. Anic-Milosevic S, Lapter-Varga M, Slaj M. Analysis of the soft tissue facial profile of Croatians using of linear measurements. J Craniofac Surg. 2008b;19: 251-258.

4. Bao B, Yu S, Tan J, Cai Y, Tian W, Ye X, et al. The analysis of frontal facial soft tissue of normal native adult of han race of Guangdong province by using the computer assisted photogrammetric-system. Hua Xi Kou Qiang Yi Xue Za Zhi. 1997;15: 266-268.

5. Choe K, Sclafani A, Litner J, Yu G, Romo TI. The Korean American woman's face: anthropometric measurements and quantitative analysis of facial aesthetics. Arch Facial Plast Surg. 2004;6: 244-252.

6. Etöz B, Etöz A, Ercan I. Nasal shapes and related differences in nostril forms: a morphometric analysis in young adults. J Craniofac Surg. 2008;19: 1402-1408.

7. Fernández-Riveiro P, Suárez-Quintanilla D, Smyth-Chamosa E, Suárez-Cunqueiro M. Linear photogrammetric analysis of the soft tissue facial profile. Am J Orthod Dentofacial Orthop. 2002;122: 59-66.

8. Gode S, Tiris F, Akyildiz S, Apaydin F. Photogrammetric analysis of soft tissue facial profile in Turkish rhinoplasty population. Aesthetic Plast Surg. 2011;35: 1016-1021.

9. Husein OF, Sepehr A, Garg R, Sina-Khadiv M, Gattu S, Waltzman J, et al. Anthropometric and aesthetic analysis of the Indian American woman's face. J Plast Reconstr Aesthet Surg. 2010;63: 1825-1831.

10. Lee D, Kim W, Chung C, Kim S, Baek S. Photogrammetric study on the face of adult Korean female. J Korean Soc Plast Reconstr Surg. 1989;16: 423-432.

11. Mostafa A, Banu L, Sultana A. Lower Jaw and Orolabial Analysis in Adult Bangladeshi Buddhist Chakma Females. Chattagram Maa-O-Shishu Hospital Med College J. 2013;12: 5-8.

12. Osunwoke E, Omin E. Photometric facial analysis of soft tissue profile of Okrika adults. Annu Res Rev Biol. 2014;4: 1980-1987.

13. Ozdemir ST, Sigirli D, Ercan I, Cankur NS. Photographic facial soft tissue analysis of healthy Turkish young adults: anthropometric measurements. Aesthetic Plast Surg. 2009;33: 175-184.

14. Porter J, Olson K. Anthropometric facial analysis of the African American woman. Arch Facial Plast Surg. 2001;3: 191-197.

15. Porter J. The average African American male face: an anthropometric analysis. Arch Facial Plast Surg. 2004;6: 78-81.

16. Sepehr A, Mathew PJ, Pepper JP, Karimi K, Devcic Z, Karam AM. The Persian woman's face: a photogrammetric analysis. Aesthetic Plast Surg. 2012;36: 687-691.

17. Song W, Koh K, Kim S, Hu K, Kim H, Park J, et al. Horizontal angular asymmetry of the face in korean young adults with reference to the eye and mouth. J Oral Maxillofac Surg. 2007;65: 2164-2168.

18. Yoo J, Kim J, Shin K, Kim S, Choi H, Jeon H, et al. Centralization or decentralization of facial structures in Korean young adults. J Craniofac Surg. 2013;24: 1007-1010.
