# Supplementary material for: Inter-Ethnic/Racial Facial Variations: A Systematic Review and Bayesian Meta-Analysis of Photogrammetric Studies
Source: PLoS One. 2015 Aug 6;10(8):e0134525. doi: 10.1371/journal.pone.0134525 (PMC4527668; doi:10.1371/journal.pone.0134525)
Supplement: S5 Table — (DOCX) [file pone.0134525.s007.docx]

**S8 Table. Risk of bias of included studies.**

| **Criterion** | | | **Akhter et al., 2013 [1]** | **Anibor et al., 2010a [2]** | **Anibor et al., 2010b [3]** | **Anibor et al., 2011 [4]** | **Anic´-Miloševic et al., 2008 a [5], b [6]** | **Bao et al., 1997 [7]** | **Chiu et al., 1992 [8]** | **Choe et al., 2004 [9]** | **Eliakim-Ikechukwu et al., 2013 (Ibo Nigerian) [10]** | **Eliakim-Ikechukwu et al., 2013 (Yoruba Nigerian) [10]** |
| --- | --- | --- | --- | --- | --- | --- | --- | --- | --- | --- | --- | --- |
| **Ⅰ. Study design** | | |  |  |  |  |  |  |  |  |  |  |
|  | A. | Objective clearly formulated | 0 | 0 | 0 | 0 | 0 | 0 | 0 | 0 | 0 | 0 |
|  | B. | Sample size for each gender ≥ 30 subjects | 0 | 0 | 0 | 0 | 0 | 0 | 0.5 | 0 | 0 | 0 |
|  | C. | Sampling method clearly reported | 1 | 1 | 1 | 1 | 0.5 | 1 | 0.5 | 0.5 | 1 | 1 |
|  | D. | Inclusion criteria clearly reported | 1 | 0 | 0 | 0 | 0 | 0 | 0 | 0.5 | 0.5 | 0.5 |
| **Ⅱ. Photo taking process** | | |  |  |  |  |  |  |  |  |  |  |
|  | E. | Subjects' body posture clearly reported | 0.5 | 1 | 0.5 | 1 | 0 | 0 | 0 | 1 | 0.5 | 0.5 |
|  | F. | Subjects' head position clearly reported | 1 | 0 | 0 | 1 | 0 | 0.5 | 0 | 1 | 0.5 | 0.5 |
|  | G. | Subjects' occlusal position clearly reported | 1 | 1 | 1 | 1 | 1 | 0 | 0 | 1 | 1 | 1 |
|  | H. | Subjects' lip posture clearly reported | 1 | 1 | 1 | 1 | 0 | 0 | 0 | 1 | 1 | 1 |
|  | I. | Camera-subject distance clearly reported | 0 | 1 | 1 | 1 | 0 | 0.5 | 0 | 1 | 0 | 0 |
|  | J. | Photographic parameters clearly reported | 1 | 1 | 1 | 1 | 0.5 | 1 | 0 | 1 | 1 | 1 |
| **Ⅲ. Facial measurements** | | |  |  |  |  |  |  |  |  |  |  |
|  | K. | Definitions of landmarks clearly described | 0.5 | 1 | 0.5 | 1 | 0.5 | 0.5 | 0 | 0.5 | 1 | 1 |
|  | L. | Definitions of linear measurements clearly described | 0 | .. | .. | .. | 0 | 0 | 0 | 0 | .. | .. |
|  | M. | Definitions of angular measurements clearly described | .. | 0 | 0 | 0 | 0 | 0 | 0 | 0.5 | 0 | 0 |
|  | N. | Attempts to ensure and quantify reliability | 1 | 1 | 1 | 1 | 0 | 1 | 0 | 1 | 1 | 1 |
| **Ⅳ. Statistical analysis** | | |  |  |  |  |  |  |  |  |  |  |
|  | O. | Statistical analysis appropriate for data | 0 | 0 | 0 | 0 | 0 | 0 | 0 | 0 | 0 | 0 |
|  | P. | Confounders accounted for in analysis | 0 | 0 | 0 | 0 | 0 | 0 | 0 | 0 | 0 | 0 |
|  | Q. | Confidence intervals provided | 0 | 0 | 0 | 0 | 0 | 0 | 0 | 0 | 0 | 0 |
| **Percentage score** | | | 0.5 | 0.5 | 0.44 | 0.56 | 0.15 | 0.26 | 0.06 | 0.53 | 0.47 | 0.47 |
| **Risk of bias level** | | | H | H | H | H | L | L | L | H | H | H |

(cont’d)

| **Criterion** | | | **Etöz et al., 2008 [11]** | **Ferdousi et al., 2013 [12]** | **Fernandez-Riveiro et al., 2002 [13]** | **Fernández-Riveiro et al., 2003 [14]** | **Gode et al., 2011 [15]** | **He et al., 2009 [16]** | **Husein et al., 2010 [17]** | **Kale-Varlk, 2008 [18]** | **Lee et al., 1989 [19]** | **Lin et al., 2013 [20]** |
| --- | --- | --- | --- | --- | --- | --- | --- | --- | --- | --- | --- | --- |
| **Ⅰ. Study design** | | |  |  |  |  |  |  |  |  |  |  |
|  | A. | Objective clearly formulated | 0 | 0 | 0 | 0 | 0 | 0 | 0 | 0 | 0 | 0 |
|  | B. | Sample size for each gender ≥ 30 subjects | 0 | 0 | 0 | 0 | 1 | 0 | 0 | 0 | 0 | 0 |
|  | C. | Sampling method clearly reported | 0.5 | 1 | 0.5 | 0.5 | 1 | 0.5 | 1 | 1 | 1 | 0.5 |
|  | D. | Inclusion criteria clearly reported | 0.5 | 0.5 | 1 | 0.5 | 0.5 | 0 | 0.5 | 0 | 0.5 | 0 |
| **Ⅱ. Photo taking process** | | |  |  |  |  |  |  |  |  |  |  |
|  | E. | Subjects' body posture clearly reported | 1 | 0.5 | 0 | 0 | 0.5 | 1 | 1 | 0 | 0 | 0 |
|  | F. | Subjects' head position clearly reported | 1 | 0 | 0 | 0 | 1 | 0.5 | 1 | 0 | 0.5 | 1 |
|  | G. | Subjects' occlusal position clearly reported | 1 | 1 | 1 | 1 | 1 | 1 | 1 | 0 | 1 | 1 |
|  | H. | Subjects' lip posture clearly reported | 1 | 1 | 0 | 0 | 0.5 | 1 | 1 | 0 | 1 | 1 |
|  | I. | Camera-subject distance clearly reported | 1 | 0.5 | 1 | 1 | 0 | 0 | 1 | 0 | 0 | 0 |
|  | J. | Photographic parameters clearly reported | 0.5 | 0.5 | 0 | 0 | 0.5 | 0.5 | 1 | 0.5 | 0.5 | 1 |
| **Ⅲ. Facial measurements** | | |  |  |  |  |  |  |  |  |  |  |
|  | K. | Definitions of landmarks clearly described | 0 | 0.5 | 0.5 | 0.5 | 0.5 | 0 | 0.5 | 0.5 | 0.5 | 0 |
|  | L. | Definitions of linear measurements clearly described | 0 | .. | 0 | .. | 0 | .. | 0 | .. | 0 | .. |
|  | M. | Definitions of angular measurements clearly described | .. | 0 | .. | 0 | 0 | 0.5 | 0.5 | 0 | 0.5 | 0 |
|  | N. | Attempts to ensure and quantify reliability | 1 | 0.5 | 0 | 0 | 1 | 0.5 | 1 | 0 | 1 | 0.5 |
| **Ⅳ. Statistical analysis** | | |  |  |  |  |  |  |  |  |  |  |
|  | O. | Statistical analysis appropriate for data | 0 | 0 | 0 | 0 | 0 | 0 | 0 | 0 | 0 | 0 |
|  | P. | Confounders accounted for in analysis | 0 | 0 | 0 | 0 | 0 | 0 | 0 | 0 | 0 | 0 |
|  | Q. | Confidence intervals provided | 0 | 0 | 0 | 0 | 0 | 0 | 0 | 0 | 0 | 0 |
| **Percentage score** | | | 0.53 | 0.38 | 0.25 | 0.22 | 0.44 | 0.34 | 0.56 | 0.13 | 0.38 | 0.31 |
| **Risk of bias level** | | | H | L | L | L | H | L | H | L | L | L |

(cont’d)

| **Criterion** | | | **Loveday et al., 2011 [21]** | **Malkoç et al., 2009 [22]** | **Mostafa, et al., 2013 (25-35 year old group) [23]** | **Mostafa, et al., 2013 (35-45 year old group) [23]** | **Oghenemavwe et al., 2010 [24]** | **Osunwoke et al., 2014 [25]** | **Ozdemir et al., 2009 [26]** | **Porter et al., 2001 [27]** | **Porter, 2004 [28]** | **Reddy et al., 2011 [29]** |
| --- | --- | --- | --- | --- | --- | --- | --- | --- | --- | --- | --- | --- |
| **Ⅰ. Study design** | | |  |  |  |  |  |  |  |  |  |  |
|  | A. | Objective clearly formulated | 0 | 0 | 0 | 0 | 0 | 0 | 0 | 0 | 0 | 0 |
|  | B. | Sample size for each gender ≥ 30 subjects | 0 | 0 | 0 | 0 | 0 | 0 | 0 | 0 | 0 | 0 |
|  | C. | Sampling method clearly reported | 0.5 | 0.5 | 0.5 | 0.5 | 0.5 | 0.5 | 0.5 | 0.5 | 0.5 | 0.5 |
|  | D. | Inclusion criteria clearly reported | 0.5 | 0 | 0 | 0 | 0.5 | 0.5 | 0.5 | 0.5 | 0.5 | 0 |
| **Ⅱ. Photo taking process** | | |  |  |  |  |  |  |  |  |  |  |
|  | E. | Subjects' body posture clearly reported | 1 | 0 | 1 | 1 | 0 | 1 | 0 | 1 | 1 | 0 |
|  | F. | Subjects' head position clearly reported | 0 | 0 | 1 | 1 | 0 | 0 | 0 | 1 | 1 | 0 |
|  | G. | Subjects' occlusal position clearly reported | 1 | 1 | 1 | 1 | 1 | 1 | 1 | 1 | 1 | 1 |
|  | H. | Subjects' lip posture clearly reported | 0 | 0 | 0.5 | 0.5 | 0 | 0 | 0.5 | 1 | 1 | 0 |
|  | I. | Camera-subject distance clearly reported | 0 | 1 | 0 | 0 | 1 | 0 | 0 | 1 | 1 | 1 |
|  | J. | Photographic parameters clearly reported | 0.5 | 0 | 0.5 | 0.5 | 0.5 | 1 | 0.5 | 1 | 1 | 0 |
| **Ⅲ. Facial measurements** | | |  |  |  |  |  |  |  |  |  |  |
|  | K. | Definitions of landmarks clearly described | 1 | 0.5 | 0.5 | 0.5 | 0.5 | 0.5 | 0 | 0.5 | 0.5 | 0.5 |
|  | L. | Definitions of linear measurements clearly described | 0 | .. | 0 | 0 | 0 | 0 | 0 | 0 | 0 | .. |
|  | M. | Definitions of angular measurements clearly described | 0 | 0 | .. | .. | 0 | 0 | .. | 0.5 | 0.5 | 0 |
|  | N. | Attempts to ensure and quantify reliability | 1 | 0 | 0.5 | 0.5 | 1 | 1 | 0 | 1 | 1 | 0 |
| **Ⅳ. Statistical analysis** | | |  |  |  |  |  |  |  |  |  |  |
|  | O. | Statistical analysis appropriate for data | 0 | 0 | 0 | 0 | 0 | 0 | 0 | 0 | 0 | 0 |
|  | P. | Confounders accounted for in analysis | 0 | 0 | 0 | 0 | 0 | 0 | 0 | 0 | 0 | 0 |
|  | Q. | Confidence intervals provided | 0 | 0 | 0 | 0 | 0 | 0 | 0 | 0 | 0 | 0 |
| **Percentage score** | | | 0.32 | 0.19 | 0.34 | 0.34 | 0.29 | 0.32 | 0.19 | 0.53 | 0.53 | 0.19 |
| **Risk of bias level** | | | L | L | L | L | L | L | L | H | H | L |

(cont’d)

| **Criterion** | | | **Sepehr et al., 2012 [30]** | **Sim et al., 2000 [31]** | **Song et al., 2007 [32]** | **Ukoha et al., 2012 [33]** | **Wamalwa et al., 2011 (Chinese) [34]** | **Wamalwa et al., 2011 (Kenyan) [34]** | **Wang et al., 2009 [35]** | **Yoo et al., 2013 [36]** |
| --- | --- | --- | --- | --- | --- | --- | --- | --- | --- | --- |
| **Ⅰ. Study design** | | |  |  |  |  |  |  |  |  |
|  | A. | Objective clearly formulated | 0 | 0 | 0 | 0 | 0 | 0 | 0 | 0 |
|  | B. | Sample size for each gender ≥ 30 subjects | 0 | 0 | 0 | 0 | 0 | 0 | 1 | 0 |
|  | C. | Sampling method clearly reported | 0.5 | 1 | 0.5 | 0.5 | 1 | 1 | 1 | 0.5 |
|  | D. | Inclusion criteria clearly reported | 0.5 | 0.5 | 0.5 | 0.5 | 0 | 0 | 0.5 | 0.5 |
| **Ⅱ. Photo taking process** | | |  |  |  |  |  |  |  |  |
|  | E. | Subjects' body posture clearly reported | 1 | 1 | 0.5 | 1 | 0 | 0 | 1 | 0.5 |
|  | F. | Subjects' head position clearly reported | 1 | 1 | 0.5 | 0 | 0 | 0 | 1 | 0.5 |
|  | G. | Subjects' occlusal position clearly reported | 1 | 1 | 1 | 1 | 1 | 1 | 1 | 1 |
|  | H. | Subjects' lip posture clearly reported | 1 | 1 | 1 | 0 | 0 | 0 | 1 | 1 |
|  | I. | Camera-subject distance clearly reported | 1 | 1 | 0 | 0.5 | 0 | 0 | 1 | 0 |
|  | J. | Photographic parameters clearly reported | 0.5 | 0.5 | 0.5 | 0.5 | 0 | 0 | 1 | 0.5 |
| **Ⅲ. Facial measurements** | | |  |  |  |  |  |  |  |  |
|  | K. | Definitions of landmarks clearly described | 0.5 | 0.5 | 0.5 | 0 | 0 | 0 | 0.5 | 0.5 |
|  | L. | Definitions of linear measurements clearly described | 0 | 0 | 0 | 0 | .. | .. | .. | 0 |
|  | M. | Definitions of angular measurements clearly described | .. | 0.5 | .. | 0 | 0 | 0 | 0.5 | .. |
|  | N. | Attempts to ensure and quantify reliability | 1 | 1 | 0.5 | 1 | 0 | 0 | 1 | 1 |
| **Ⅳ. Statistical analysis** | | |  |  |  |  |  |  |  |  |
|  | O. | Statistical analysis appropriate for data | 0 | 0 | 0 | 0 | 0 | 0 | 0 | 0 |
|  | P. | Confounders accounted for in analysis | 0 | 0 | 0 | 0 | 0 | 0 | 0 | 0 |
|  | Q. | Confidence intervals provided | 0 | 0 | 0 | 0 | 0 | 0 | 0 | 0 |
| **Percentage score** | | | 0.5 | 0.53 | 0.34 | 0.29 | 0.13 | 0.13 | 0.66 | 0.38 |
| **Risk of bias level** | | | H | H | L | L | L | L | H | L |

..: Inapplicable. L: low risk of bias. H: high risk of bias.

**References**

1. Akhter Z, Banu M, Alam M, Hossain S, Nazneen M. Photo-anthropometric study on face among Garo adult females of Bangladesh. Bangladesh Med Res Counc Bull. 2013;39: 61-64.

2. Anibor E. Photometric facial analysis of the Ibo ethnic group in Nigeria. Arch Appl Sci Res. 2010a;2: 219-222.

3. Anibor E. Photometric facial analysis of the Urhobo ethnic group in Nigeria. Arch Appl Sci Res. 2010b;2: 28-32.

4. Anibor E. Photometric facial analysis of the Itsekiri ethnic group in Nigeria. Adv Appl Sci Res. 2011;2: 145-148.

5. Anic-Milosevic S, Lapter-Varga M, Slaj M. Analysis of the soft tissue facial profile by means of angular measurements. Eur J Orthod. 2008a;30: 135-140.

6. Anic-Milosevic S, Lapter-Varga M, Slaj M. Analysis of the soft tissue facial profile of Croatians using of linear measurements. J Craniofac Surg. 2008b;19: 251-258.

7. Bao B, Yu S, Tan J, Cai Y, Tian W, Ye X, et al. The analysis of frontal facial soft tissue of normal native adult of han race of Guangdong province by using the computer assisted photogrammetric-system. Hua Xi Kou Qiang Yi Xue Za Zhi. 1997;15: 266-268.

8. Chiu C, Clark R. The facial soft tissue profile of the southern Chinese: prosthodontic considerations. J Prosthet Dent. 1992;68: 839-850.

9. Choe K, Sclafani A, Litner J, Yu G, Romo TI. The Korean American woman's face: anthropometric measurements and quantitative analysis of facial aesthetics. Arch Facial Plast Surg. 2004;6: 244-252.

10. Eliakim-Ikechukwu C, Ekpo A, Etika M, Ihentuge C, Mesembe O. Facial aesthetic angles of the Ibo and Yoruba ethnic groups of Nigeria. IOSR J Pharm Biol Sci. 2013;5: 14-17.

11. Etöz B, Etöz A, Ercan I. Nasal shapes and related differences in nostril forms: a morphometric analysis in young adults. J Craniofac Surg. 2008;19: 1402-1408.

12. Ferdousi M, Mamun A, Banu L, Paul S. Angular Photogrammetric Analysis of the Facial Profile of the Adult Bangladeshi Garo. Adv Anthropol. 2013;3: 188-192.

13. Fernández-Riveiro P, Suárez-Quintanilla D, Smyth-Chamosa E, Suárez-Cunqueiro M. Linear photogrammetric analysis of the soft tissue facial profile. Am J Orthod Dentofacial Orthop. 2002;122: 59-66.

14. Fernández-Riveiro P, Smyth-Chamosa E, Suárez-Quintanilla D, Suárez-Cunqueiro M: Angular photogrammetric analysis of the soft tissue facial profile. Eur J Orthod. 2003;25: 393-399.

15. Gode S, Tiris F, Akyildiz S, Apaydin F. Photogrammetric analysis of soft tissue facial profile in Turkish rhinoplasty population. Aesthetic Plast Surg. 2011;35: 1016-1021.

16. He Z, Jian X, Wu X, Gao X, Zhou S, Zhong X. Anthropometric measurement and analysis of the external nasal soft tissue in 119 young Han Chinese adults. J Craniofac Surg. 2009;20: 1347-1351.

17. Husein OF, Sepehr A, Garg R, Sina-Khadiv M, Gattu S, Waltzman J, et al. Anthropometric and aesthetic analysis of the Indian American woman's face. J Plast Reconstr Aesthet Surg. 2010;63: 1825-1831.

18. Kale-Varlk S. Angular photogrammetric analysis of the soft tissue facial profile of Anatolian Turkish adults. J Craniofac Surg. 2008;19: 1481-1486.

19. Lee D, Kim W, Chung C, Kim S, Baek S. Photogrammetric study on the face of adult Korean female. J Korean Soc Plast Reconstr Surg. 1989;16: 423-432.

20. Lin C, Shaari R, Alam M, Rahman S. Photogrammetric Analysis of Nasolabial Angle and Mentolabial Angle norm in Malaysian Adults. Bangladesh J Med Sci. 2013;12: 209-214.

21. Loveday O, Babatunde F, Isobo U, Sunday O, Ijeoma O. Photogrammetric analysis of soft tissue profile of the face of Igbos in Port Harcourt. Asian J Med Sci. 2011;3: 228-233.

22. Malkoc S, Demir A, Uysal T, Canbuldu N. Angular photogrammetric analysis of the soft tissue facial profile of Turkish adults. Eur J Orthod. 2009;31: 174-179.

23. Mostafa A, Banu L, Sultana A. Lower Jaw and Orolabial Analysis in Adult Bangladeshi Buddhist Chakma Females. Chattagram Maa-O-Shishu Hospital Med College J. 2013;12: 5-8.

24. Oghenemavwe E, Osunwoke A, Ordu S, Omovigho O. Photometric analysis of soft tissue facial profile of adult Urhobos. Asian J Med Sci. 2010;2: 248-252.

25. Osunwoke E, Omin E. Photometric facial analysis of soft tissue profile of Okrika adults. Annu Res Rev Biol. 2014;4: 1980-1987.

26. Ozdemir ST, Sigirli D, Ercan I, Cankur NS. Photographic facial soft tissue analysis of healthy Turkish young adults: anthropometric measurements. Aesthetic Plast Surg. 2009;33: 175-184.

27. Porter J, Olson K. Anthropometric facial analysis of the African American woman. Arch Facial Plast Surg. 2001;3: 191-197.

28. Porter J. The average African American male face: an anthropometric analysis. Arch Facial Plast Surg. 2004;6: 78-81.

29. Reddy M, Ahuja N, Raghav P, Kundu V, Mishra V. Computer-assisted angular photogrammetric analysis of the soft tissue facial profile of North Indian adults. J Indian Orthod Soc. 2011;45: 119-123.

30. Sepehr A, Mathew PJ, Pepper JP, Karimi K, Devcic Z, Karam AM. The Persian woman's face: a photogrammetric analysis. Aesthetic Plast Surg. 2012;36: 687-691.

31. Sim R, Smith J, Chan A. Comparison of the aesthetic facial proportions of southern Chinese and white women. Arch Facial Plast Surg. 2000;2: 113-120.

32. Song W, Koh K, Kim S, Hu K, Kim H, Park J, et al. Horizontal angular asymmetry of the face in korean young adults with reference to the eye and mouth. J Oral Maxillofac Surg. 2007;65: 2164-2168.

33. Ukoha U, Udemezue O, Oranusi C, Asomugha A, Dimkpa U, Nzeukwu L. Photometric facial analysis of the Igbo Nigerian adult male. Niger Med J. 2012;53: 240-244.

34. Wamalwa P, Amisi SK, Wang Y, Chen S. Angular photogrammetric comparison of the soft-tissue facial profile of Kenyans and Chinese. J Craniofac Surg. 2011;22: 1064-1072.

35. Wang J, Jang Y, Park S, Lee B. Measurement of aesthetic proportions in the profile view of Koreans. Ann Plast Surg. 2009;62: 109-113.

36. Yoo J, Kim J, Shin K, Kim S, Choi H, Jeon H, et al. Centralization or decentralization of facial structures in Korean young adults. J Craniofac Surg. 2013;24: 1007-1010.
