# Supplementary material for: Inter-Ethnic/Racial Facial Variations: A Systematic Review and Bayesian Meta-Analysis of Photogrammetric Studies
Source: PLoS One. 2015 Aug 6;10(8):e0134525. doi: 10.1371/journal.pone.0134525 (PMC4527668; doi:10.1371/journal.pone.0134525)
Supplement: S6 Table — (DOCX) [file pone.0134525.s008.docx]

**S6 Table. Criteria for risk of bias assessment.**

| **Criterion** | | | **Free from bias (0)** | **Partially free from bias (0.5)** | **Subject to bias (1)** |
| --- | --- | --- | --- | --- | --- |
| **Ⅰ. Study design** | | |  |  |  |
|  | A. | Objective clearly formulated | Clearly formulated | ·· | Not clearly formulated |
|  | B. | Sample size for each gender ≥ 30 subjects | Sample size by gender ≥ 30 | Sample size for one gender < 30, and ≥ 30 for the other gender (applicable to studies with both genders) | Sample size by gender < 30 |
|  | C. | Sampling method clearly reported | Sampling method reported with details | Sampling method reported without details | Sampling method not reported |
|  | D. | Inclusion criteria clearly reported | General health status and occlusal traits reported | General health status or occlusal traits reported | Not reported |
| **Ⅱ. Photo taking process** | | |  |  |  |
|  | E. | Subjects' body posture clearly reported | Subjects were standing during photographing | Subjects were seated during photographing | Not reported |
|  | F. | Subjects' head position clearly reported | NHP | Head positions other than NHP | Not reported |
|  | G. | Subjects' occlusal position clearly reported | Centric occlusion | ·· | Not reported |
|  | H. | Subjects' lip posture clearly reported | Relaxed lip | Closed lip | Not reported |
|  | I. | Camera-subject distance clearly reported | Exact distance reported | Approximate distance reported | Not reported |
|  | J. | Photographic parameters clearly reported | Camera, lens, shutter speed, and aperture described | Camera, lens, shutter speed, or aperture described | Not reported |
| **Ⅲ. Facial measurements** | | |  |  |  |
|  | K. | Definitions of landmarks clearly described | Written definitions provided | Illustrated with photos | Not reported |
|  | L. | Definitions of linear measurements clearly described | Defined using landmarks | Illustrated with photos | Not reported |
|  | M. | Definitions of angular measurements clearly described | Defined using landmarks | Illustrated with photos | Not reported |
|  | N. | Attempts to ensure and quantify reliability | Attempts made to ensure reliability, and method error reported | Attempts made to ensure reliability, but method error not reported | Not reported |
| **Ⅳ. Statistical analysis** | | |  |  |  |
|  | O. | Statistical analysis appropriate for data | Appropriate method of analysis | ·· | Inappropriate method of analysis |
|  | P. | Confounders accounted for in analysis | Confounders considered | ·· | Confounders not considered |
|  | Q. | Confidence intervals provided | Provided or could be estimated | ·· | Not provided and could not be estimated |

··: Inapplicable.
