# Supplementary material for: Inter-Ethnic/Racial Facial Variations: A Systematic Review and Bayesian Meta-Analysis of Photogrammetric Studies
Source: PLoS One. 2015 Aug 6;10(8):e0134525. doi: 10.1371/journal.pone.0134525 (PMC4527668; doi:10.1371/journal.pone.0134525)
Supplement: S1 Text — (DOCX) [file pone.0134525.s009.docx]

**S1 Text. Protocol of the systematic review** **(May 2014).**

**TITLE**

Inter-ethnic/racial facial variations: a systematic review and Bayesian meta-analysis of photogrammetric studies

**REVIEW TEAM**

Yi Feng Wen, Hai Ming Wong, Ruitao Lin, Guosheng Yin, Colman McGrath

**BACKGROUND**

Establishing normative values of facial measurements is a prerequisite for determining the degree of deviations from normality in patients with congenital anomalies and post-traumatic disfigurements. During diagnosis and treatment of such craniofacial disfigurements, variations of the facial features among ethnic/racial groups must be considered. Despite their clinical importance, normative values and inter-ethnic/racial variations of facial measurements remain under-investigated.

The most comprehensive study to date investigating inter-ethnic/racial facial variations comes from Farkas’ study where direct anthropometric method was applied to subjects from 25 countries [1]. However, only 30 subjects were recruited by gender from each country, and the generalizability of this study was therefore largely compromised. Another study by Fang and colleagues [2] systematically reviewed inter-ethnic/racial variations of linear facial measurements based on seven anthropometric studies. Noting that facial measurements obtained through photogrammetric and direct anthropometric methods were pooled together for analysis by Fang and colleagues, bias might have been introduced into the synthesized results [3].

Photogrammetry has been advocated for large epidemiological studies investigating soft tissue facial measurements because of its time saving nature and the widespread availability of the equipment [4]. Increasingly, photogrammetric studies around the world are providing normative values for facial features.

**AIMS & OBJECTIVES**

The aim of this systematic review is to establish database for normative values of facial measurements at ethnicity/race level and to explore inter-ethnic/racial facial variations based on photogrammetric studies.

Specific objectives of this study are:

1. To estimate ethnicity/race-specific normative values of 11 angular and 18 linear measurements;
2. To identify significant inter-ethnic/racial variations of the facial measurements.

**METHODS**

**Search strategy**

*Electronic databases*

The following electronic databases will be searched

- PubMed (1997 onward)
- ISI Web of Science (1956 onward)
- EMBASE (1947 onward)
- Scopus (1995 onward)

Dates of initial search will be recorded. Regular e-mail alerts from electronic databases will be received to keep the search updated.

*Search terms*

The search terms will be developed using controlled vocabularies, e.g. MeSH terms in PubMed, and keywords. Three groups of words will constitute the search strategy: (1) the method by which the measurements were taken (photogrammetry); (2) the body area of interest (the face); and (3) the measurements of interest (linear and angular measurements).

*Hand searches*

Reference lists of all eligible studies will be accessed to identify other potentially eligible studies.

**First round of screening: title and abstract screening**

A comprehensive search of the four electronic databases will be performed using the pre-defined search strategy and the records retrieved will be imported to the EndNote software. Duplications will then be removed using the “Find Duplicates” function in EndNote. Any additional records identified through hand search will be added to EndNote library manually.

Titles and abstracts of identified records will be screened by two trained and calibrated reviewers (YFW and HMW) and the records will then be classified into one of the following categories using predefined inclusion and exclusion criteria:

- Potentially eligible, full-text will be accessed
- Exclude
- Unclear

Discrepant opinions between the two reviewers will be resolved by discussion and further consultation of a third reviewer (CM). Reasons for exclusion will be documented.

**Second round of screening: full-text screening**

Full-texts of potentially eligible studies and those “Unclear” studies will be accessed and screened in the same way as in the first round. Reasons for exclusion will be documented.

Inter-reviewer agreement on study selection will be assessed using the $\kappa$ statistics for both round of screening.

**Study selection**

*Inclusion criteria*

Eligible studies should meet all of the following criteria

- Subjects’ age: 18-45 years old
- Ethnicity/Race: ethnicity/race of the subjects should be either African, Asian or Caucasian according to Risch and colleagues’ classification scheme [5]
- Definitions of the measurements: the landmarks and facial measurements should be defined in line with the publicly accepted definitions proposed by Farkas and other researchers [6-8]
- Reporting of results: standard error could be estimated from the reported values; the reported values should be accurate to one decimal place
- Language: no restrictions were imposed
- Date: no restrictions were imposed
- Status of publication: no restrictions were imposed

*Exclusion criteria*

Studies will be excluded if they focus exclusively on

- Attractive/beautiful subjects
- Subjects with severe malocclusion, developmental craniofacial disfigurement, history of facial trauma/fracture and cosmetic surgery
- Patients with systematic disorders known to affect craniofacial development

Studies will also be excluded if they are reviews, abstracts, or conference proceedings.

**Data extraction**

Data will be extracted using a computerized data extraction sheet developed in Microsoft Excel 2013. The sheet will be pilot-tested prior to its implementation by comparing the data extracted by three independent reviewers (YFW, HMW and CM) on the basis of 5 randomly selected studies. Any ambiguities in the sheet will be discussed and the sheet amended accordingly. The data will be extracted by one reviewer (YFW) and verified by another reviewer (HMW). Discrepant opinions will be resolved by discussion or consultation of a third reviewer (CM).

The following data will be extracted

- Study identifiers: author(s), year of publication
- Study characteristics: study location, sample size, source of sample
- Demographics: ethnicity/race of subjects, subjects’ age in years, occlusal trait of the subjects
- Details of the photographic process: body position, head posture, occlusion position, and lip/chin posture during photo-taking, camera-subject distance
- Facial measurements: angular and linear facial measurements of interest will be extracted (selection of the measurements will be based on their clinical importance and availability from eligible studies)

**Assessment of Study Quality**

Risk of bias of eligible studies will be assessed by two independent reviewers (YFW and HMW) based an instrument [9] that has been used in similar systematic reviews on anthropometric studies [10,11]. Further modifications of the instrument are made to fit for the purpose of this review. This instrument assesses four domains of eligible studies:

- Study design
- Photo taking process
- Facial measurements
- Appropriateness of statistical analysis

A score of 0, 0.5 or 1 will be assigned to each item indicating free of bias, partially free of bias and subject to bias, respectively. Inapplicable items will not be scored. A percentage score will be calculated for each study by dividing the sum of item scores by the total number of applicable items. The lower the score of a study, the lower the risk that its findings will be biased. A score of 0.40 will be used as the cut-off value to differentiate studies with high and low risk of bias.

**Data analysis**

Pertaining to the aim of this review, the extracted data will be organised into a hierarchical structure in which individual studies are nested within ethnicities/races that in turn are nested within the total population. We will apply a Bayesian hierarchical random effects model to accommodate this data structure to allow the information to exchange across ethnicities/races.

Let $y_{ij}$ denote the observed value of a facial measurement from the $i^{th}$ study of the $j^{th}$ ethnicity/race. Our model is given as below:

$$y_{ij}=\theta_{ij}+\epsilon_{ij}, \epsilon_{ij}\sim N\left( 0,v_{ij}^{2} \right), \left( 1 \right)$$

­­$\theta_{ij}=\delta_{0j}+\zeta_{ij}, \zeta_{ij}\sim N\left( 0,\sigma^{2} \right), \left( 2 \right)$

$$\delta_{0j}=\mu_{00}+\eta_{0j}, \eta_{0j}\sim N\left( 0,\tau^{2} \right). \left( 3 \right)$$

$$\left( 1 \right)+\left( 2 \right)+\left( 3 \right)$$

$$y_{ij}=\mu_{00}+\eta_{0j}+\zeta_{ij}+\epsilon_{ij}. (4)$$

where $y_{ij}$ is assumed to be independently and normally distributed with mean $\theta_{ij}$ and variance $v_{ij}^{2}$. The error term $\epsilon_{ij}$ is normally distributed with a mean of zero and a variance $v_{ij}^{2}$. In the second level of the hierarchy, each study’s true effect size $\theta_{ij}$ varies around an ethnicity/race mean $\delta_{0j}$. The study-specific random effect $\zeta_{ij}$ is normally distributed with a mean of zero and variance $\sigma^{2}$. At the third level of the hierarchy, the ethnicity/race means vary around an overall mean $\mu_{00}$ and the ethnicity/race-specific random effect $\eta_{0j}$ is normally distributed with a mean of zero and variance $\tau^{2}$.

Half-Cauchy prior distribution was assigned to $\sigma$ and $\tau$ with the scale set to be 25, namely

$$f\left( \sigma\right) = \frac{\pi}{2}\frac{25}{\sigma^{2}+{25}^{2}}, \sigma>0 (5)$$

$$f\left( \tau\right) = \frac{\pi}{2}\frac{25}{\tau^{2}+{25}^{2}}. \tau>0 (6)$$

To estimate inter-ethnic/racial variability of the facial measurements, linear contrasts are constructed as below:

$$\delta_{12}= \delta_{02}-\delta_{01}, (7)$$

$$\delta_{13}= \delta_{03}-\delta_{01}, (8)$$

$$\delta_{23}= \delta_{03}-\delta_{02}. (9)$$

where $\delta_{01}$, $\delta_{02}$, and $\delta_{03}$ represent posterior means of the facial measurements for Africans, Asians and Caucasians, respectively.

The model will be fitted using the Markov chain Monte Carlo (MCMC) algorithm in R [12] and JAGS [13] through the R-package R2jags [14]. Convergence of the MCMC algorithm and the sufficiency of the number of effectively independent posterior draws will be checked by visual inspection of the trace plots and the convergence diagnostic potential scale reduction factor [15].

Posterior means and the corresponding 95% credible intervals (CrIs) of the estimates will be used to establish population norms of the facial measurements at the ethnicity/race level. Significance of inter-ethnic/racial variations of the facial measurements will be explored by examining whether 0 was included in 95% and 90% CrIs of the linear contrasts, corresponding to a significance level of 0.05 and 0.10, respectively.

**References**

1. Farkas LG, Katic MJ, Forrest CR, Alt KW, Bagic I, Baltadjiev G, et al. International anthropometric study of facial morphology in various ethnic groups/races. J Craniofac Surg. 2005;16: 615-646.

2. Fang F, Clapham P, Chung K. A systematic review of interethnic variability in facial dimensions. Plast Reconstr Surg. 2011;127: 874-881.

3. Higgins J, Thompson S, Deeks J, Altman D. Statistical heterogeneity in systematic reviews of clinical trials: a critical appraisal of guidelines and practice. J Health Serv Res Policy. 2002;7: 51-61.

4. Zhang X, Hans M, Graham G, Kirchner H, Redline S. Correlations between cephalometric and facial photographic measurements of craniofacial form. Am J Orthod Dentofacial Orthop. 2007;131: 67-71.

5. Risch N, Burchard E, Ziv E, Tang H. Categorization of humans in biomedical research: genes, race and disease. Genome Biol. 2002;3: comment2007.

6. Farkas LG. Anthropometry of the Head and Face. 2nd ed. New York: Raven Press, Ltd.; 1994.

7. Naini FB. Facial Aesthetics: Concepts and Clinical Diagnosis. West Sussex, UK: Wiley-Blackwell; 2011.

8. Powell N, Humphreus B. Proportions of the aesthetic face. New York: Thieme-Stratton Inc.; 1984.

9. Gordon JM, Rosenblatt M, Witmans M, Carey JP, Heo G, Major PW, et al. Rapid palatal expansion effects on nasal airway dimensions as measured by acoustic rhinometry. A systematic review. Angle Orthod. 2009;79: 1000-1007.

10. Brons S, van Beusichem M, Bronkhorst E, Draaisma J, Bergé S, Schols J, et al. Methods to quantify soft tissue-based cranial growth and treatment outcomes in children: a systematic review. PLoS One. 2014;9: e89602.

11. Brons S, van Beusichem M, Bronkhorst E, Draaisma J, Bergé S, Maal T, et al. Methods to quantify soft-tissue based facial growth and treatment outcomes in children: a systematic review. PLoS One. 2012;7: e41898.

12. R Development Core Team: R: A Language and Environment for Statistical Computing [http://www.R-project.org]

13. Plummer M. JAGS: A program for analysis of Bayesian graphical models using Gibbs sampling In: Proceedings of the 3rd International Workshop on Distributed Statistical Computing (DSC 2003): 2003; Vienna, Austria; 2003.

14. Su Y, Yajima M. R2jags: A Package for Running jags from R [http://cran.r-project.org/web/packages/R2jags/index.html]

15. Gelman A, Rubin D. Inference from iterative simulation using multiple sequences. Stat Sci. 1992;7: 457-472.
