# Supplementary material for: Inter-Ethnic/Racial Facial Variations: A Systematic Review and Bayesian Meta-Analysis of Photogrammetric Studies
Source: PLoS One. 2015 Aug 6;10(8):e0134525. doi: 10.1371/journal.pone.0134525 (PMC4527668; doi:10.1371/journal.pone.0134525)
Supplement: S2 Text — (DOCX) [file pone.0134525.s010.docx]

# S2 Text. Meta-analysis of Observational Studies in Epidemiology (MOOSE) Checklist.

**Inter-ethnic/racial facial variations: a systematic review and Bayesian meta-analysis of photogrammetric studies**

**Review team**

Dr. Yi Feng Wen

Dr. Hai Ming Wong

Paediatric Dentistry & Orthodontics, Faculty of Dentistry, The University of Hong Kong, Hospital Road, Hong Kong

Dr. Ruitao Lin

Prof. Guosheng Yin

Department of Statistics & Actuarial Science, Faculty of Science, The University of Hong Kong, Pokfulam Road, Hong Kong

Prof. Colman McGrath

Periodontology & Public Health, Faculty of Dentistry, The University of Hong Kong, Hospital Road, Hong Kong

| **Criteria** | | **Brief description of how the criteria were handled in the meta-analysis** |
| --- | --- | --- |
| **Reporting of background should include** | |  |
| √ | Problem definition | Results from different anthropometric methods are not directly comparable. To date, no meta-analysis of photogrammetric studies has been performed. |
| √ | Hypothesis statement | Inter-ethnic/racial facial variations exist for angular and linear measurements. |
| √ | Description of study outcomes | Establish database for population norms of various angular and linear facial measurements for Africans, Asians and Caucasians; and to determine inter-ethnic/racial facial variations. |
| √ | Type of exposure or intervention used | Photogrammetry. |
| √ | Type of study designs used | This review sought to identify all facial photogrammetric studies regardless of the type of study design. |
| √ | Study population | Subjects of eligible studies were either Africans, Asians or Caucasians. |
| **Reporting of search strategy should include** | |  |
| √ | Qualifications of searchers | The credentials of the three investigators YFW, HMW, and CM are indicated in the author list. |
| √ | Search strategy, including time period included in the synthesis and keywords | Search strategy: Additional file 3: Document S3.  The initial search was updated to 1^st^ December, 2014 using automatic e-mail alerts. |
| √ | Effort to include all available studies | We comprehensively searched the electronic databases with no restrictions on language, dates or status of publication. Reference lists of articles that were identified in the screening process were also manually searched. |
| √ | Databases and registries searched | PubMed (1997 onward), ISI Web of Science (1956 onward), EMBASE (1947 onward) and Scopus (1995 onward) |
| √ | Search software used, name and version, including special features used | No search software was used. EndNote was used to manage retrieved records and remove duplications. |
| √ | Use of hand searching | Reference lists of articles that were identified in the screening process were also manually searched. |
| √ | List of citations located and those excluded, including justification | Details of the literature search process are outlined in the Figure 1. Reasons for exclusion were also recorded in Figure 1. |
| √ | Method of addressing articles published in languages other than English | We placed no restrictions on language. One study was in Chinese, one in Korean, and the remaining 36 studies were in English. |
| √ | Method of handling abstracts and unpublished studies | Abstracts were excluded. We did not find unpublished photogrammetric studies. |
| √ | Description of any contact with authors | We attempted to acquire missing information by E-mail enquiry of the studies’ correspondence author whenever needed. |
| **Reporting of methods should include** | |  |
| √ | Description of relevance or appropriateness of studies assembled for assessing the hypothesis to be tested | Detailed inclusion and exclusion criteria were described in the Methods section under the subheading Study selection. |
| √ | Rationale for the selection and coding of data | Data extracted from each of the studies were relevant to study characteristics and demographics, photographic process and data for meta-analysis. |
| √ | Assessment of confounding | Our meta-analysis inherits the limitations of original research. We did not adjust our analyses for other anthropometric indices such as body weight, height or body mass index since they were reported in none of the eligible studies. |
| √ | Assessment of study quality, including blinding of quality assessors; stratification or regression on possible predictors of study results | Risk of bias was assessed based on an instrument that has been used in systematic reviews on craniofacial anthropometrics. A score of 0, 0.5 or 1 was assigned to each item indicating free of bias, partially free of bias and subject to bias, respectively. Studies with average scores below 0.40 were considered as with low risk of bias. |
| √ | Assessment of heterogeneity | Heterogeneity of the studies were indicated by the varying risk of bias scores, which ranged from 0.06 to 0.66. |
| √ | Description of statistical methods in sufficient detail to be replicated | Descriptions of method of meta-analyses are introduced in Methods section and described in detail in Additional file 10: Document S4. |
| √ | Provision of appropriate tables and graphics | We included tables for landmark and measurement definition, extracted measurements, risk of bias assessment, study characteristics and demographics, and ethnicity/race-specific estimates. Figures illustrating the study selection procedure and highlight key findings of the study are provided. |
| **Reporting of results should include** | |  |
| √ | Graph summarizing individual study estimates and overall estimate | Figures 3 |
| √ | Table giving descriptive information for each study included | Table 1 |
| √ | Results of sensitivity testing (eg, subgroup analysis) | Studies were categorized into 3 ethnic/racial groups, linear contrasts were constructed to explore inter-ethnic/racial facial variations. |
| √ | Indication of statistical uncertainty of findings | Estimates of the facial measurements were informed by posterior means and 95% credible intervals. Inter-ethnic/racial variations were examined at significance levels of 0.05 and 0.10. |
| **Reporting of discussion should include** | |  |
| √ | Quantitative assessment of bias | A percentage score indicating risk of bias was calculated for each study. Each item in the risk of bias assessment instrument was also evaluated. |
| √ | Justification for exclusion | We excluded the studies if they failed to meet the inclusion criteria. |
| √ | Assessment of quality of included studies | Risk of bias of the studies differed and a notably high percentage of African studies (58.3%) were with high risk of bias. |
| **Reporting of conclusions should include** | |  |
| √ | Consideration of alternative explanations for observed results | We discussed that we did not adjust our analyses for other anthropometric indices since they were reported in none of the eligible studies. In addition, we discussed that estimates derived from small amount of data may be subject to bias when applied to the population at large. |
| √ | Generalization of the conclusions | The results can provide a useful resource to guide research and clinical practice. |
| √ | Guidelines for future research | This study also highlights the need for more high quality photogrammetric studies employing standardized photographic techniques; and preferably from a large randomized sample comprising different ethnic/racial groups. |
| √ | Disclosure of funding source | The work described in this paper was fully supported by a grant from the Research Grants Council of the Hong Kong Special Administrative Region, China (Project No. 781112). |
