# Supplementary material for: Inter-Ethnic/Racial Facial Variations: A Systematic Review and Bayesian Meta-Analysis of Photogrammetric Studies
Source: PLoS One. 2015 Aug 6;10(8):e0134525. doi: 10.1371/journal.pone.0134525 (PMC4527668; doi:10.1371/journal.pone.0134525)
Supplement: S3 Text — (DOCX) [file pone.0134525.s011.docx]

**S3 Text. Detailed search strategy.**

**PubMed (1997 onward)**

1. Photogram [tiab]
2. photogrammetry [majr]
3. photogrammetr* [tiab]
4. photograph* [tiab]
5. anthropometry [majr]
6. anthropometr* [tiab]
7. morphometr* [tiab]
8. 1 OR 2 OR 3 OR 4 OR 5 OR 6 OR 7
9. face [majr]
10. face [tiab]
11. facies [majr]
12. facies [tiab]
13. facial [tiab]
14. facial profile [tiab]
15. craniofacial [tiab]
16. 9 OR 10 OR 11 OR 12 OR 13 OR 14 OR 15
17. linear measurements [tiab]
18. angles [tiab]
19. angular measurements [tiab]
20. proportion indices [tiab]
21. esthetics [majr]
22. esthetics [tiab]
23. aesthetics [tiab]
24. esthetic proportions [tiab]
25. 17 OR 18 OR 19 OR 20 OR 21 OR 22 OR 23 OR 24
26. 8 AND 16 AND 25

**Embase (1947 onward)**

1. Photogram.tw.
2. photogrammetr*.tw.
3. photograph.tw.
4. photography/
5. exp anthropoetry/
6. exp morphometrics/
7. 1 OR 2 OR 3 OR 4 OR 5 OR 6
8. face/
9. craniofacial.tw.
10. facial profile.mp.
11. 8 OR 9 OR 10
12. linear measurements.tw.
13. angles.mp.
14. angular measurements.tw.
15. proportion indices.tw.
16. exp esthetics/
17. aesthetic proportions.mp.
18. 12 OR 13 OR 14 OR 15 OR 16 OR 17
19. 7 AND 11 AND 18

**Scopus (1995 onward)**

1. TITLE-ABS-KEY(photogram)
2. TITLE-ABS-KEY(photogrammetr*)
3. TITLE-ABS-KEY(photograph*)
4. TITLE-ABS-KEY(anthropometr*)
5. TITLE-ABS-KEY(morphometr*)
6. 1 OR 2 OR 3 OR 4 OR 5 OR
7. TITLE-ABS-KEY(face)
8. TITLE-ABS-KEY(facies)
9. TITLE-ABS-KEY(facial)
10. TITLE-ABS-KEY(facial profile)
11. OR TITLE-ABS-KEY(craniofacial)
12. 7 OR 8 OR 9 OR 10 OR 11
13. TITLE-ABS-KEY(linear measurements)
14. TITLE-ABS-KEY(angles)
15. TITLE-ABS-KEY(angular measurements)
16. TITLE-ABS-KEY(proportion indices)
17. TITLE-ABS-KEY(aesthetic)
18. TITLE-ABS-KEY(aesthetic proportions)
19. 13 OR 14 OR 15 OR 16 OR 17 OR 18
20. 6 AND 12 AND 19

**ISI Web of Science (1956 onward)**

1. TOPIC: (photogram)
2. TOPIC: (photogrammetr*)
3. TOPIC: (photograph*)
4. TOPIC: (anthropometr*)
5. TOPIC: (morphometr*)
6. 1 OR 2 OR 3 OR 4 OR 5
7. TOPIC: (face)
8. TOPIC: (facies)
9. TOPIC: (facial)
10. TOPIC: (facial profile)
11. TOPIC: (craniofacial)
12. 7 OR 8 OR 9 OR 10 OR 11
13. TOPIC: (linear measurements)
14. TOPIC: (angles)
15. TOPIC: (angular measurements)
16. TOPIC: (proportion indices)
17. TOPIC: (aesthetics)
18. TOPIC: (aesthetic proportions)
19. 13 OR 14 OR 15 OR 16 OR 17 OR 18
20. 6 AND 12 AND 19
