# Supplementary material for: Inter-Ethnic/Racial Facial Variations: A Systematic Review and Bayesian Meta-Analysis of Photogrammetric Studies
Source: PLoS One. 2015 Aug 6;10(8):e0134525. doi: 10.1371/journal.pone.0134525 (PMC4527668; doi:10.1371/journal.pone.0134525)
Supplement: S4 Text — (DOCX) [file pone.0134525.s012.docx]

**S4 Text. Specification of the Bayesian hierarchical random effects model.**

**1 Data**

Standard deviation ($SD_{ij}$) and sample size ($n_{ij}$) of the $i^{th}$ eligible study of the $j^{th}$ ethnicity/race were extracted and used to estimate standard error ($SE_{ij}$) using the formula $SE_{ij} =SD_{ij}/\sqrt{n_{ij}}$. If, however, confidence interval ($CI_{ij}$) was reported instead of $SD_{ij}$, $SE_{ij}$ was estimated as $((upper bound of CI_{ij}) -lower bound of CI_{ij}) )/(2\times1.96)$. Sampling variance $v_{ij}^{2}$ was finally obtained through the equation $v_{ij}^{2} = SE_{ij}^{2}$.

**2 Bayesian hierarchical model**

Our dataset has a hierarchical structure in which individual studies were nested within ethnicities/races that in turn were nested within the total population. This data structure provides particular benefits to the application of Bayesian hierarchical random effects model in that it allows the model to borrow strength to compromise between complete pooling, in which the grand estimates are overly simplified, and no pooling, in which within-unit estimates are overly noisy [1]. The information is adaptively pooled across different studies using Bayesian hierarchical modelling, to a greater degree when data are sparse and to a lesser extent when data are rich.

**2.1 Three-level meta-analytic model**

Flow diagram and path diagram illustrating the hierarchical structure is provided in S1 and S2 Figs. Let $y_{ij}$ denote the observed value of a facial measurement from the $i^{th}$ study of the $j^{th}$ ethnicity/race. We assume $y_{ij}$ to be independently and normally distributed with mean $\theta_{ij}$ and variance $v_{ij}^{2}$. Hence the within-study model describing the first level of the hierarchy is

$$y_{ij}=\theta_{ij}+\epsilon_{ij}, \epsilon_{ij}\sim N\left( 0,v_{ij}^{2} \right), \left( 1 \right)$$

where the error term $\epsilon_{ij}$ is normally distributed with mean 0 and sampling variance $v_{ij}^{2}$. In the second level of the hierarchy where the true effect size $\theta_{ij}$ varies around the ethnicity/race mean $\delta_{0j}$, the between-study within-ethnicity/race model is

$$\theta_{ij}=\delta_{0j}+\zeta_{ij}, \zeta_{ij}\sim N\left( 0,\sigma^{2} \right), \left( 2 \right)$$

where $\zeta_{ij}$ is a study-specific random effect that is normally distributed with mean 0 and between-study variance $\sigma^{2}$. Finally, at the third level the ethnicity/race means vary around an overall mean $\mu_{00}$ and the between-ethnicity/race model is

$$\delta_{0j}=\mu_{00}+\eta_{0j}, \eta_{0j}\sim N\left( 0,\tau^{2} \right), \left( 3 \right)$$

where $\eta_{0j}$ is an ethnicity/race-specific random effect that is normally distributed with mean 0 and between-ethnicity/race variance $\tau^{2}$. The overall model can be written as

$$y_{ij}=\mu_{00}+\eta_{0j}+\zeta_{ij}+\epsilon_{ij}. (4)$$

We allow heterogeneity of the variances of sampling errors $v_{ij}^{2}$ in the within-study model, while the variances of random effects $\zeta_{ij}$ and $\eta_{0j}$ are assumed homogeneous in the between-study within-ethnicity/race and between-ethnicity/race model, respectively. In addition, since the units at each level of the hierarchy are independently distributed, the error terms $\epsilon_{ij}$, $\zeta_{ij}$ and $\eta_{0j}$ are therefore uncorrelated.

**2.2 Prior distributions**

Since the number of ethnic/racial groups $J$ in our dataset is small (below 5), the uniform prior distribution tends to overestimate the variance parameter $\sigma^{2}$ and $\tau^{2}$ [2]. On the other hand, the non-informative inverse-gamma distribution has been criticized as prior distribution for variance parameters in that the resulting inferences tend to be sensitive to selection of the shape and scale parameters [2]. Following Gelman’s recommendation, we chose the half-Cauchy prior distribution for $\sigma$ and $\tau$ with the scale set to be 25 ^2^, namely

$$f\left( \sigma\right) = \frac{\pi}{2}\frac{25}{\sigma^{2}+{25}^{2}}, \sigma>0 (5)$$

$$f\left( \tau\right) = \frac{\pi}{2}\frac{25}{\tau^{2}+{25}^{2}}. \tau>0 (6)$$

The overall mean $\mu_{00}$ was assigned a non-informative normal prior $\mu_{00}\sim N({0,10}^{4})$.

**2.3 Linear contrast**

To estimate inter-ethnic/racial variation of the facial measurements, linear contrasts are constructed as below

$$\delta_{12}= \delta_{02}-\delta_{01}, (7)$$

$$\delta_{13}= \delta_{03}-\delta_{01}, (8)$$

$$\delta_{23}= \delta_{03}-\delta_{02}. (9)$$

where $\delta_{01}$, $\delta_{02}$, and $\delta_{03}$ represent posterior means of the facial measurements for Africans, Asians and Caucasians, respectively.

1. **Computation**

We fitted the Bayesian hierarchical model using the Markov chain Monte Carlo (MCMC) algorithm with R [3] and JAGS [4] applying the R-package R2jags [5]. To allow for exchange of information between- and within-ethnicities/races such that more stable and reliable estimates can be obtained, facial measurements were meta-analysed only if there were data from two or three ethnicities/races when at least one of the ethnicities/races were informed by two or more eligible studies. We implemented three Markov chains in parallel using randomly selected initial values. After a burn-in of 50000 iterations, each chain was ran for another 50000 iterations. Combining the three chains and thinning by a factor of ten, we finally obtained 15000 posterior samples from which we obtained the results and drew statistical inferences. Convergence of the MCMC algorithm and the sufficiency of the number of effectively independent posterior draws were checked by visual inspection of the trace plots and the convergence diagnostic potential scale reduction factor [6].

**4 Inference**

Database for normative values of the facial measurements was established at the ethnicity/race level by extracting posterior means and the corresponding 95% credible intervals (CrIs) of $\delta_{0k}$. Inter-ethnic/racial variations of the facial measurements were considered significant if 0 was not included in the 95% and 90% CrIs of the linear contrasts, corresponding to a significance level of 0.05 and 0.10, respectively.

**References**

1. Gelman A, Hill J. Data analysis using regression and multilevel/hierarchical models. Cambridge, UK: Cambridge University Press; 2007.

2. Gelman A. Prior distributions for variance parameters in hierarchical models (Comment on Article by Browne and Draper). Bayesian Anal. 2006;1: 515-34.

3. R Development Core Team. R: A Language and Environment for Statistical Computing [http://www.R-project.org]

4. Plummer M. JAGS: A program for analysis of Bayesian graphical models using Gibbs sampling In: Proceedings of the 3rd International Workshop on Distributed Statistical Computing (DSC 2003): 2003; Vienna, Austria; 2003.

5. Su Y, Yajima M. R2jags: A Package for Running jags from R [http://cran.r-project.org/web/packages/R2jags/index.html]

6. Gelman A, Rubin D. Inference from iterative simulation using multiple sequences. Stat Sci. 1992;7: 457-472.
